# Analyzing Acceptor-like State Distribution of Solution-Processed Indium-Zinc-Oxide Semiconductor Depending on the In Concentration

**DOI:** 10.3390/nano13152165

**Published:** 2023-07-26

**Authors:** Dongwook Kim, Hyeonju Lee, Youngjun Yun, Jaehoon Park, Xue Zhang, Jin-Hyuk Bae, Sungkeun Baang

**Affiliations:** 1 School of Information Science, Hallym University, Chuncheon 24252, Republic of Korea; d.kim@hallym.ac.kr (D.K.); hyeonjulee@hallym.ac.kr (H.L.); jaypark@hallym.ac.kr (J.P.); 2 School of Nano Convergence Technology, Hallym University, Chuncheon 24252, Republic of Korea; youngjun.yun@hallym.ac.kr; 3 Department of Electronic Engineering, Hallym University, Chuncheon 24252, Republic of Korea; 4 College of Ocean Science and Engineering, Shangdong University of Science and Technology, Qingdao 266590, China; skd996027@sdust.edu.cn; 5 School of Electronics Engineering, Kyungpook National University, Daegu 41566, Republic of Korea; 6 School of Electronic and Electrical Engineering, Kyungpook National University, Daegu 41566, Republic of Korea

**Keywords:** density of state, solution-processed IZO semiconductor, thin-film transistor, thermal analysis

## Abstract

Understanding the density of state (DOS) distribution in solution-processed indium-zinc-oxide (IZO) thin-film transistors (TFTs) is crucial for addressing electrical instability. This paper presents quantitative calculations of the acceptor-like state distribution of solution-processed IZO TFTs using thermal energy analysis. To extract the acceptor-like state distribution, the electrical characteristics of IZO TFTs with various In molarity ratios were analyzed with respect to temperature. An Arrhenius plot was used to determine electrical parameters such as the activation energy, flat band energy, and flat band voltage. Two calculation methods, the simplified charge approximation and the Meyer–Neldel (MN) rule-based carrier–surface potential field-effect analysis, were proposed to estimate the acceptor-like state distribution. The simplified charge approximation established the modeling of acceptor-like states using the charge–voltage relationship. The MN rule-based field-effect analysis validated the DOS distribution through the carrier–surface potential relationship. In addition, this study introduces practical and effective approaches for determining the DOS distribution of solution-processed IZO semiconductors based on the In molarity ratio. The profiles of the acceptor-like state distribution provide insights into the electrical behavior depending on the doping concentration of the solution-processed IZO semiconductors.

## 1. Introduction

Solution-processed indium-zinc-oxide (IZO) metal-oxide semiconductors have emerged as promising materials for a wide range of electronic applications, including transparent conductive films, thin-film transistors (TFTs), and photovoltaic devices [1,2,3,4]. These metal-oxide semiconductors exhibit desirable properties such as high field-effect mobility, optical transparency, and the potential for direct printing, making them ideal for next-generation electronic devices [5,6,7,8]. However, a comprehensive understanding of their electronic behavior and the underlying factors derived from their material properties is essential for optimizing their properties and addressing their electrical instability [9,10,11].

Almost every electrical characteristic of TFT operation is closely related to the density of state (DOS) distribution of semiconductor materials [11,12]. TFTs operate via charge accumulation induced in the semiconductor channel. This induced charge can be explained by band bending at the semiconductor interface and the occupied acceptor-like states. When the energy band changes owing to external energy or applied voltage, the amount of accumulated charge varies, leading to changes in the drain current and the threshold voltage V_Th_.

**Figure 1:**
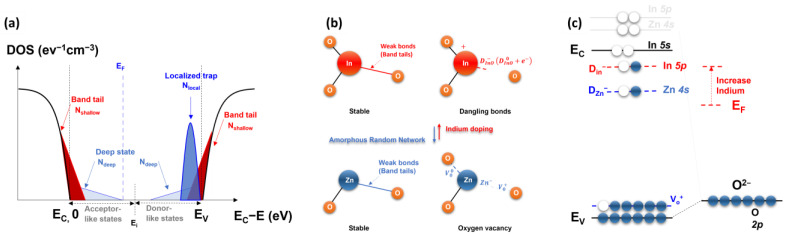
Schematic illustrating (**a**) the band gap state of the amorphous IZO semiconductor, (**b**) the atomic bonding structure of the amorphous random network in the IZO semiconductor, and (**c**) the energy band diagram of the IZO semiconductor, including the band gap states.

Figure 1 shows a representative DOS distribution model for the IZO semiconductor, the atomic bonding structure model of the IZO semiconductor material, and the band diagram of the IZO semiconductor. Based on the DOS model depicted in Figure 1a, the direct relationship between the TFT current characteristics and the semiconductor DOS profile can be addressed. As shown in Figure 1a, the surface charge density Qs can be estimated by integrating the product of the DOS distribution, which is distributed exponentially or according to a Gaussian function, and the Fermi–Dirac function. The TFT current can be defined as a function of the channel charge Q_s_ and the drain voltage. Conversely, when a model for the bandgap state is not defined, the DOS distribution can be extracted by observing the changes in the drain current with respect to the external energy. Because of the significantly lower hole mobility compared to electrons in n-type semiconductors such as IZO TFTs, estimating the DOS distribution towards the valence band under the Fermi energy E_F_ is difficult. However, the distribution of acceptor-like states above E_F_ can be easily analyzed using thermal energy.

As mentioned above, research on the acceptor-like states of semiconductor materials has primarily been discussed using carrier transport models with respect to temperature. Previous studies estimated the distribution of the density of states near and at the conduction band edge of semiconductor materials by analyzing the variation in carrier mobility with temperature. Representative mobility models, the hopping transport theory for organic semiconductors [13,14,15,16], the multiple trapping and release (MTR) theory for a-Si:H [17,18], and the percolation theory for metal-oxide semiconductors [19,20] depict the distribution of acceptor-like states near the conductor band edge based on charge transport models. In addition to the carrier mobility model, research has been conducted to directly calculate the acceptor-like state distribution from the current–voltage characteristics of TFTs based on thermal energy [21,22,23,24]. In these studies, the distribution of acceptor-like states near the conduction band edge was estimated by analyzing the changes in electrical parameters caused by thermal energy. Furthermore, in another study, the distribution of acceptor-like states was determined based on the transition characteristics of trapping/detrapping charges with thermal energy [25]. 

In the case of the solution-processed IZO semiconductor, as shown in Figure 1b, the atomic bonding structure changes with the In molar ratio, resulting in shifts in the energy band diagram as depicted in Figure 1c. These changes occur owing to an increase in weak bonds, dangling bonds, and oxygen vacancies in the In–O atomic bonding, which consequently leads to an increase in the donors. Consequently, the distribution of the energy states and the position of the Fermi energy E_F_ shift. Because the electrical conductivity and threshold voltage of IZO TFTs are closely associated with these doping characteristics, the doping effect, which depends on the In molarity ratio and the quantitative extraction of the DOS distribution, is crucial for solution-processed IZO TFTs. Furthermore, a precise understanding of the DOS distribution can facilitate the interpretation of the electrical instability caused by charge trapping/detrapping in solution-processed IZO TFTs.

In this study, we quantitatively calculated the distribution of acceptor-like states in solution-processed IZO TFTs using thermal analysis. To determine the distribution of acceptor-like states, we analyzed the transfer characteristics of TFTs with different In molarity ratios and extracted the DOS distribution using two calculation methods. An Arrhenius plot was constructed based on the transfer curves with respect to temperature, and electrical parameters such as activation energy, flat band energy, and flat band voltage were extracted and analyzed for each In molarity ratio. Among the proposed methods for DOS calculation, the simplified charge approximation method models the acceptor-like state by determining the channel charge–gate voltage relationship under given thermal energy conditions. In the Meyer–Neldel (MN) rule-based carrier–surface potential field-effect analysis, the applied thermal energy condition was characterized using the MN parameter, and the DOS distribution was verified using carrier–surface potential functions. Furthermore, this study proposes practical and effective methods for calculating the acceptor-like state distribution of solution-processed IZO TFTs with respect to the In molarity ratio. 

## 2. Materials and Methods

**Figure 2:**
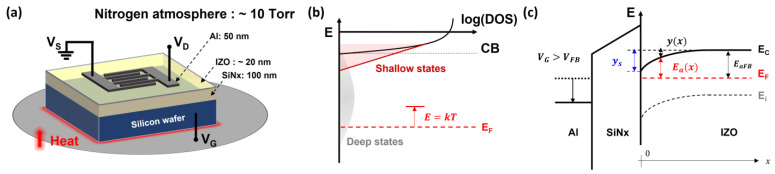
(**a**) Schematic illustration depicting the device structure of a solution-processed IZO TFT and the measurement atmosphere of the vacuum chamber. (**b**) Acceptor-like state distribution of an IZO semiconductor. (**c**) Energy band diagram of a metal-gate dielectric-IZO semiconductor illustrating the key energy levels.

To extract semiconductor acceptor-like states, we employed solution-processed IZO TFTs, as shown in Figure 2a. The IZO solution used to fabricate the TFTs was prepared by dissolving indium nitrate hydrate (In_3_(NO_3_)_3_∙xH_2_O) and zinc nitrate hydrate (Zn_2_(NO_3_)_2_∙xH_2_O) in 2-methoxyethanol (CH_3_O(CH_2_)_2_OH), also known as the 2-ME solvent. The In:Zn molar ratios used in the preparation of the IZO solutions are listed in Table 1. The prepared solution, according to the ratios listed in Table 1, was spin-coated onto a p-type Si wafer with a sputtered 100 nm thick SiN_x_ gate dielectric. The resulting coating formed an IZO semiconductor layer with a thickness of approximately 20 nm. The source/drain electrodes were fabricated through thermal deposition, and the finger-type structure had a W/L ratio of 2000 μm/80 μm. The detailed fabrication process for the electrical characteristics of solution-processed IZO TFTs can be found in previous studies [26,27]. 

The electrical characteristics of the TFTs with respect to temperature were efficiently controlled and measured using a vacuum chamber probe station (M56VC) (MS Tech., Kyoungki, Republic of Korea). Most oxide semiconductor devices are susceptible to exposure to air at high temperatures. Thus, the nitrogen gas atmosphere within the chamber was maintained at approximately 10 Torr. The temperature of the TFT substrate was increased up to 270 °C from room temperature (RT) using a halogen lamp, and most devices experienced breakdown above 270 °C. The electrical characteristics of solution-processed IZO TFTs were measured using a semiconductor analyzer (Keithly-4200A) (Tektronix, Beaverton, OR, USA), and the transfer characteristics at saturation current were swept from gate voltage V_G_ = −20 V to 40 V with a drain voltage V_D_ = 20 V. The transfer characteristics of all devices were measured up to 240 °C from RT at 5 °C intervals, and the temperature was increased at a rate of 5 °C per min. 

## 3. Simple Charge Approximation

Electrons that are induced or excited by gate voltage or thermal energy at the Fermi energy level exhibit free transport within the conduction band of the IZO semiconductor. Similarly, when electrons are filled (occupied) in acceptor-like states within the semiconductor band gap due to the surface bending or the thermal energy, the probability of these electrons being present on the conduction band increases, thereby affecting the electrical conductivity of the IZO TFT [28,29,30,31]. The change in charge corresponding to variations in gate voltage can be defined by Q = CV_G_, and the distribution of acceptor-like states over the Fermi energy, E_F_, corresponds to the number of excited carriers per unit of induced energy. The calculation method in this study for the acceptor-like state distribution is based on the approach proposed by Lang et al., which extracts the DOS from the relationship between the gate voltage and excited carrier density [32,33,34]. 

Figure 2 depicts the (a) fabricated TFT, (b) DOS distribution of the IZO semiconductor, and (c) energy band diagram. In Figure 2b, the total DOS distribution for the acceptor-like states is the sum of the shallow and deep states N_a_(E) = N_shallow_(E) + N_deep_(E), and the induced free carrier density n(E) can be expressed as the integral of the product of the acceptor-like state and the Fermi–Dirac function:

(1) $$n\left(E\right)=\int_{E_{F}}^{E}N_{a}\left(E\right)f\left(E\right)dE.$$

In thermal equilibrium, the variation in the free carrier density due to thermal energy can be estimated by considering the changes in the occupied states governed by the Fermi–Dirac function. Under the condition 1/N(E)·dN(E)/dE < kT and by applying the 0 K approximation, it can be observed that if E > kT, then f(E) = 0 for empty states, and if E < kT, then f(E) = 1 for occupied states. By applying the 0 K assumption, the induced thermal energy can be represented as E = kT as shown in Figure 2b. Furthermore, assuming that the induced free electrons originate from the occupied acceptor-like states N_a_(E), it can be assumed that f(E) = 1 and n(E) = ∫N(E) dE. In Figure 2c, the activation energy E_a_ represents the energy required to place electrons from E_F_ to E_C_, and by surface band bending y(x), E_a_ can be expressed as

(2) $$E_{a}\left(x\right)=E_{aFB}-y\left(x\right)$$

where y(x) is the surface band bending energy from the semiconductor–dielectric interface, and E_aFB_ is the activation energy in the flat-band configuration. The equation for the activation energy was obtained from an Arrhenius plot.

(3) $$I_{D}=I_{D0}\cdot \exp\left(-\frac{E_{a}}{kT}\right),\ln I_{D}=\ln I_{D0}-\frac{E_{a}}{kT}.$$

I_D0_ is a prefactor representing the initial ln I_D_ converging with respect to the activation energy at a certain temperature. Based on the extracted activation energy, a brief result for the semiconductor DOS can be obtained by differentiating the density of free carriers as follows:

(4) $$\frac{\partial n\left(E_{a}\right)}{\partial E_{a}}=N_{a}\left(E_{a}\right),{\left|\frac{dn\left(E_{a}\right)}{dE_{a}}\right|}_{E=kT}=N_{a}\left(E_{a}\right).$$

Assuming that V_D_ is constant under saturated conditions, the drain current of the IZO is determined by the surface potential and temperature. Thus, the drain current induced by thermal energy can be expressed as

(5) $$I_{D}\left(E\right)=-q\mu_{FE}n\left(E\right)\xi_{d}A_{DS}.$$

In this equation, q is the charge of electron, ξ_d_ denotes the electric field between the source and drain, i.e., ξ_d_ = −V_D_/L. A_DS_ is the cross-sectional area of the drain current and is given by A_DS_ = W × d_s_, where d_s_ is the channel thickness. It is assumed that the channel of the IZO TFT is sufficiently formed with a thin layer of semiconductor, approximately 20 nm in thickness. To compare the voltage-driven current characteristics with the thermal energy, the drain current of the TFT can be summarized as

(6) $$I_{D}\left(V_{G}\right)=C_{ox}\mu_{FE}\frac{W}{L}\left[\left(V_{G}-V_{Th}\right)V_{D}-\frac{1}{2}V_{D}^{2}\right]$$

where C_ox_ is the gate oxide capacitance in F/cm^2^, and the thickness of the gate dielectric, d_ox_, is 100 nm. By applying the V_D_ = V_G_ − V_Th_ for the saturation current, and considering that the equations for the drain current I_D_(E) and I_D_(V_G_) are the same, the following summary can be made:

(7) $$C_{ox}\left(V_{G}-V_{Th}\right)=2qn\left(E\right)d_{s}.$$

The equation above represents the amount of charge carried by the electrons in the channel induced by the gate voltage. This corresponds to the carriers induced by the surface band bending, and the approximate acceptor-like state under the surface band bending condition can be obtained by differentiating n(E) with respect to E_a_. It can be expressed as:

(8) $$N_{a}\left(E_{a}\right)=\frac{d}{dE_{a}}{\left[\frac{C_{OX}}{2qd_{s}}\left(V_{G}-V_{Th}\left(V_{G}\right)\right)\right]}_{E=kT}$$

**Figure 3:**
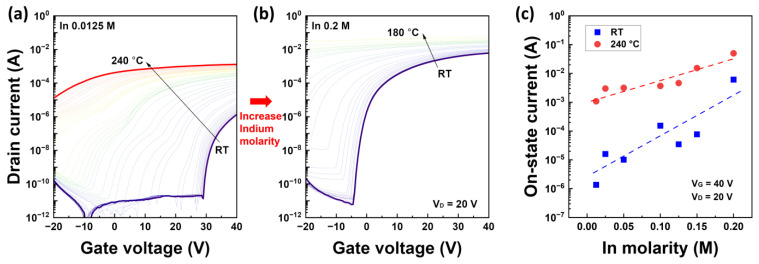
Transfer characteristics of solution-processed IZO TFT according to the measurement temperatures. (**a**) Transfer curves with 0.0125 M of In molarity and (**b**) 0.2 M of In molarity. The various colored lines in graphs (**a**,**b**) depict the results measured at temperatures ranging from RT to 240 °C. (**c**) On-state drain current as a function of In molarity ratio at RT and 240 °C.

Figure 3 shows the transfer characteristics of the IZO semiconductor as a function of temperature with respect to the In molarity ratio. Figure 3a,b show the transfer characteristics of the IZO TFTs with In molarity ratios of 0.0125 M and 0.2 M, respectively. Graph (c) illustrates the on-state current characteristics under saturation conditions at RT and 240 °C. The transfer characteristics of the IZO TFTs for each In molarity ratio are summarized in Supplementary Figure S1. In the analysis of this paper, V_D_ = 20 V was determined in the transfer curve in Figure 3, specifically in the saturation region. This was due to the relatively high leakage current of the fabricated IZO TFTs, which leads to increased external factors in the linear region [27]. Although there were some variations, the current of the IZO TFTs increased significantly with increasing temperature, and the TFT with the lowest In molarity ratio exhibited the largest increase in current. The increase in the off-state current was significantly greater than that in the on-state current. At high temperatures, control of the gate voltage is lost, resulting in only on-state characteristics. In the case of IZO TFTs with a high In molarity ratio in Figure 3b, carrier saturation is observed over the entire gate voltage range, and electric field saturation between source and drain occurs at temperatures above 160 °C, making measurements impossible. The increase in the TFT current with increasing temperature can be attributed to the carriers induced by the thermal energy and enhanced conductivity.

**Figure 4:**
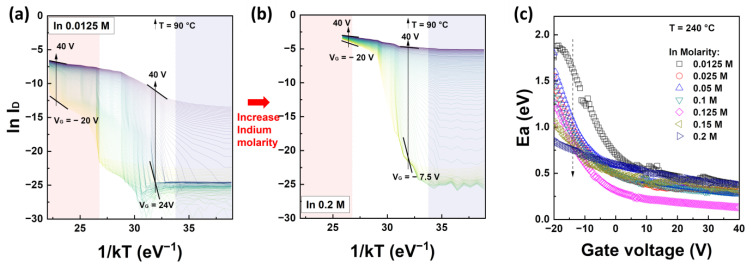
Arrhenius plots of solution-processed IZO TFTs with (**a**) 0.0125 M of In molarity ratio and (**b**) 0.2 M. The different colored lines in graphs (**a**,**b**) show the measurement results with respect to the gate voltage ranging from −20 V to 40 V. (**c**) The extracted activation energy is at T = 240 °C with respect to In molarity.

To analyze the activation energy as a function of temperature, Arrhenius plots of the IZO TFTs are presented in Figure 4a,b with respect to the In molarity ratio. More detailed Arrhenius plots for each In molarity ratio are shown in the Supplementary Materials, Figure S2. Figure 4c shows the activation energy as a function of the gate voltage extracted from the slopes of the Arrhenius plots for each In concentration. The results of the activation energy in Figure 4c were extracted at 240 °C. The Arrhenius plots in Figure 4a,b can be divided into three temperature regions: low, intermediate, and high, each exhibiting different characteristics. In the Low range, i.e., RT to 60 °C, the data varied irregularly with temperature, and negative activation energy was observed in the temperature range of 60 °C (refer to the graphs of In molarity ratio 0.05, 0.1, and 0.125 M in Figure S4). These characteristics are attributed to the trapped charges in the SiN_x_ gate dielectric, specifically the influence of the initial charge state, and it is speculated that they are activated at temperatures above 60 °C and the charges escape through the gate electrode. In the temperature range of 60 to 160 °C, the characteristics of the TFTs from the off-state to the on-state were observed overall, and the maximum variation in activation energy was observed in the range of 70 to 110 °C. Carriers induced by thermal energy or surface bending accumulate in the TFT channel starting from the flat-band condition. The flat-band voltage of the TFT can be defined as the point at which the current begins to transition from the off-state to the on-state in the Arrhenius plot. However, the measured activation energy in this range was unexpectedly much larger (approximately 8–10 times) than the theoretical background. This can be explained by the trapped charge modifying the initial conditions of the current characteristics with respect to the gate voltage under thermal equilibrium at RT [35,36,37]. This electrical behavior was alleviated, particularly at temperatures above 160 °C as shown in the red region, where the influence of gate voltage decreased. At 240 °C (kT_T=240°C_ = 0.0442 eV) in the range of −20 V to 40 V with V_D_ = 20 V, a relatively reasonable activation energy was measured, and this was defined as the activation energy by converting the data to results at 90 °C.

**Figure 5:**
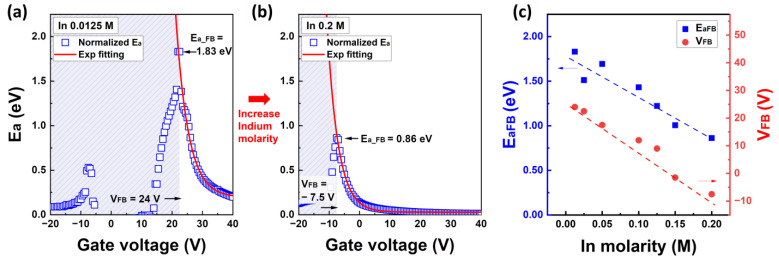
(**a**) Normalized activation energy versus gate voltage graph with 0.0125 M of In molarity and (**b**) 0.2 M of In molarity. (**c**) Activation energy and flat band voltage as a function of the In molarity ratio.

The graph in Figure 5 shows the activation energy as a function of the gate voltage according to the In concentration. Supplementary Material Figure S3 provides detailed information on the activation energy based on the molarity ratio. Figure 5a,b show the activation energy extracted at 90 °C, calculated by converting the slopes of the Arrhenius plots at 240 °C, specifically the E_a_ in Figure 4c when V_G_ = −20 V. Figure 5c illustrates the results of the extracted activation energy (E_a_) and the flat band voltage (V_FB_) values as a function of the In molarity ratio. The normalization conditions for Figure 5a,b were calculated using the following equation:

(9) $$E_{a}\left(V_{G}\right)=\frac{{E_{a}}^{\prime }{\left.\left(V_{G}\right)\right|}_{T=90^{{}^{\circ}}C}}{{\left.E_{aFB}\right|}_{T=90^{{}^{\circ}}C}}\times {\left.E_{aFB}\right|}_{T=240^{{}^{\circ}}C}.$$

The activation energy under the flat-band condition, E_aFB_, was extracted from the maximum E_a_ value in Figure 5a,b, and the corresponding gate voltage was defined as the flat-band voltage, V_FB_. The hatched region in Figure 5a,b represents the range below V_FB_ and corresponds to the off-state in the depletion region where the minimum drain current I_DS_ < 10^−13^ A. As mentioned, activation energy values deviating significantly from the expected values at T = 90 °C were normalized using the flat band energy at T = 240 °C and a gate voltage of −20 V. The E_a_ and V_FB_ characteristics of the IZO TFTs exhibit a monotonic decrease with respect to the In molar ratio as shown in Figure 5c. The inversely proportional relationship between the activation energy and gate voltage indicates that the activation energy decreases as the gate voltage increases.

**Figure 6:**
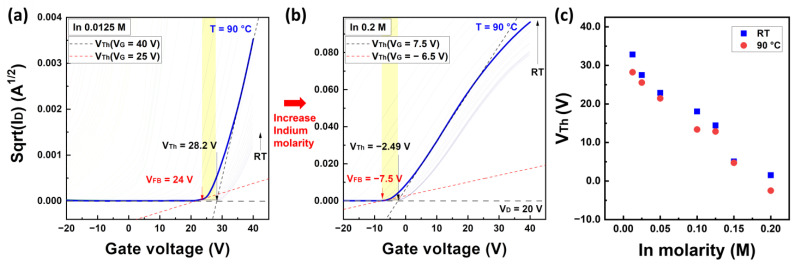
Square root of the drain current versus gate voltage for solution-processed IZO TFTs (**a**) with In 0.0125 M and (**b**) 0.2 M, where the black dashed line represents the tangent at V_G.max_, while the red dashed line represents the tangent at the gate voltage near V_FB_. The yellow region in graphs (**a**,**b**) represents the subthreshold voltage region. (**c**) The threshold voltage graph in terms of the In molarity ratio at RT and 90 °C.

To examine the relationship between the flat-band voltage and the threshold voltage, we analyzed the square root characteristics of the drain current as shown in Figure 6a,b. Figure 6c demonstrates the variation in V_Th_ with respect to the In molarity ratio, extracted under RT and T = 90 °C conditions. More detailed information on the characteristics, including the threshold voltage and field-effect mobility based on the In molarity ratio, can be found in the Supplementary Materials (Figures S4 and S5). In Figure 6a,b, the black dashed lines are extrapolated from the maximum slope of the square root graph, whereas the red dashed lines are extrapolated near the flat-band voltage. The yellow box in the figure represents the subthreshold region. Unlike single-crystal silicon-based MOSFETs, amorphous semiconductor TFTs operate under accumulated conditions without inversion. As shown in Figure 6a,b, the actual flat band voltage and threshold voltage of the IZO TFTs differed by approximately 4–5 V, regardless of the In concentration, suggesting the existence of a subthreshold region inferred from the flat band condition from the maximum flat band condition. Consequently, to describe the changes in the subthreshold voltage region more accurately, we defined the applied voltage using the gate voltage as V_F_(V_G_) = V_G_ − V_Th_(V_G_), where V_Th_(V_G_) is the extrapolated threshold voltage at each gate voltage measurement point, that is:

(10) $$V_{Th}\left(V_{G}\right)=V_{G}-\frac{\sqrt{\left(I_{D}\right)}}{g_{m}},V_{Th}=V_{G\cdot max}-\frac{\sqrt{\left(I_{D\cdot max}\right)}}{g_{m\cdot max}}.$$

where g_m_ is the transconductance of the square root of I_D_, g_m·max_ is the maximum transconductance of the square root of I_D_, I_D·max_ is the drain current at the point of g_m·max_, and V_G·max_ is the gate voltage at the point of g_m·max_, respectively. The V_Th_ characteristics at the temperatures T = RT and T = 90 °C are shown in Figure 6c, where the V_Th_ values are determined by extrapolation from the maximum transconductance. The field-effect mobility, µ_FE_, calculated from the transconductance can be found in the Supplementary Material, Figure S5. V_Th_ is approximately 1–5 V higher at T = 90 °C compared to the RT condition, and it ranges from 40% to 70% of the on-state current, regardless of the In molarity ratio. V_Th_ decreased monotonically with increasing In molarity ratio, suggesting a similar mechanism to the characteristics observed for doping in single-crystal silicon semiconductors.

**Figure 7:**
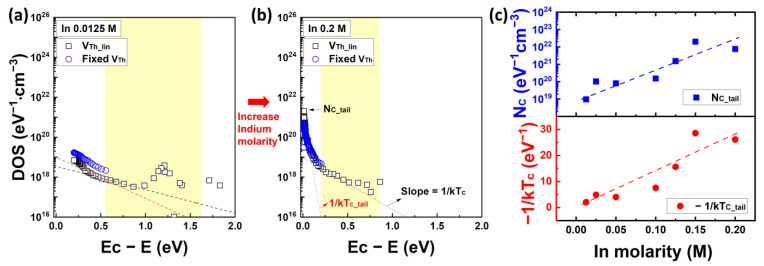
Calculated band gap state distribution and characteristic temperature of solution-processed IZO semiconductors from the conduction band to the Fermi energy level. (**a**) The DOS distribution with 0.0125 M of In molarity and (**b**) 0.2 M of In molarity. The yellow region in graphs (**a**,**b**) corresponds to the E_aFB_-E_a_, defined from the Ea extracted from the subthreshold voltage region. (**c**) The density of band tail state at the E_C_ and properties of the slope with respect to the In molarity.

Figure 7 plots the quantitative distribution of acceptor-like states calculated from Equation (8) and characteristic energy characteristics according to the In molarity ratio. Detailed information on the DOS characteristics based on the In molarity ratio can be found in the Supplementary Material (Figure S6). In particular, Figure 7c denotes a graph summarizing the DOS concentration at the conduction band edge (N_C_) and the corresponding characteristic energy kT_c_ from the exponential distribution tangent at the point N_C_ with respect to each In molarity ratio. In the graphs in Figure 7a,b, two band-tail state models are described: the shallow state (red dashed line) and the deep state (black dashed line) of the acceptor-like states. N_C_tail_ and −1/kT_c_tail_ correspond to the characteristics of the shallow state, mostly related to the In concentration. In Figure 7a,b, the black rectangles and blue circles represent the DOS distribution when using V_F_(V_G_) and fixed V_Th_ values, respectively, and the yellow box indicates the DOS distribution in the subthreshold voltage region. As depicted in Figure 7a,b, employing linear extrapolation for V_F_(V_G_) allows a more comprehensive description of the DOS profile compared to using a fixed V_Th_, including the subthreshold voltage region. The magnitude of DOS at the conduction band edge N_C_ is 9.59 × 10^18^ for a low In molarity ratio of 0.0125 M, and it increases by approximately three orders of magnitude to 7.63 × 10^21^ for a high In molarity ratio of 0.2 M. The characteristic energy kT_c_ decreases from 488 meV to approximately 38 meV, and when converted to −1/kTc, it ranges from approximately 2.05 eV^−1^ to 26.13 eV^−1^ as shown in Figure 7c.

In this study, we introduce a DOS calculation method for solution-processed IZO semiconductors using a simple charge approximation. The DOS distribution and on-state current characteristics increased exponentially with respect to the In concentration, whereas the activation energy, threshold voltage, and flat-band voltage characteristics decreased linearly. This calculation method provides insights into the extraction of the DOS distribution of solution-processed IZO semiconductors and proposes a practical approach for calculating the DOS distribution based on the In molar ratio. The advantage of this method is its fast computation based on the charge sheet approximation, which enables the approximate prediction of acceptor-like states near the conduction band. On the other hand, in order to validate the effectiveness of the simple charge approximation, we also conducted a similar analysis using the MN rule-based carrier–surface potential field-effect analysis to calculate the acceptor-like state distribution based on the current analysis.

## 4. Meyer–Neldel Rule-Based Field-Effect Analysis

MN is observed in the TFTs and is a fundamental characteristic of amorphous semiconductor materials. For the MN characteristics, there is a specific correlation between the MN parameter A and the activation energy E_a_ in the Arrhenius plot. The DOS analysis method based on the MN rule involves extracting the distribution of acceptor-like states by differentiating carriers with respect to the surface band-bending energy (y_s_) considering the applied external thermal energy condition. As defined in Equation (4), N(E) is a function of kT and y_s_, where the activation energy E_a_ is influenced by the thermal and surface energies, i.e., E_a_ = kT and E_a_(x) = E_aFB_ − y(x). Additional definitions are required to simultaneously consider thermal and surface energies as variables in a single equation. The MN parameter A, which directly correlates with the activation energy, can be used to define the influence of temperature. More detailed explanations and calculation methods for characteristics based on the MN rule are available in the literature [38,39,40,41,42,43,44,45]. The calculation method for the acceptor-like state distribution based on the MN rule used in this paper is derived from the theory proposed by C. Chen’s research group [38].

The MN prefactor I_D0_ can be calculated from the current characteristics in the Arrhenius plot using the equation ln I_DS_ = ln I_D0_ − Ea/kT, where ln I_DS_ vs. 1/kT represents the y-intercept at x = 0. The MN parameter A, defined from I_D0_, is derived from Equation (3).

(11) $$I_{D0}=I_{D00}\cdot \exp\left(A\cdot E_{a}\right),\;\;\ln I_{D0}=\ln I_{D00}+A\cdot E_{a}$$

In this equation, I_D00_ represents the MN constant. The MN parameter A is a variable determined by the temperature and y(x) is a variable influenced by the applied voltage. At low temperatures, the electrical behavior of the IZO TFTs resembled the characteristics in the subthreshold region above the flat-band voltage. At high temperatures, the electrical behavior of the IZO TFTs resembled that in the overthreshold voltage region. By determining the MN parameters in the subthreshold and overthreshold voltage regions, the thermal energy factor can be incorporated into Equation (11). Substituting Equations (3) and (11) into this relationship, the equation for the drain current as a function of the gate voltage can be obtained as follows:

(12) $$I_{D}\left(V_{G}\right)=I_{D00}\cdot \exp\left[\left(A-\beta \right)\cdot E_{a}\left(V_{G}\right)\right],\;\;\ln I_{D}\left(V_{G}\right)=\ln I_{D00}+\left(A-\beta \right)\cdot E_{a}\left(V_{G}\right)$$

The temperature-dependent factor β represents 1/kT in the equation. By substituting E_a_(x) = E_aFB_ − y(x), the drain current equation can be transformed into a function of x, as follows:

(13) $$I_{D}\left(V_{G}\right)=\frac{I_{FB}}{d_{s}}\cdot \int_{0}^{d_{s}}\exp\left[\left(\beta -A\right)\cdot y\left(x\right)\right]dx$$

where I_FB_ = I_D00_ · exp[(A − β) · E_aFB_]. To establish the relationship between the charge density and the electric field induced by the surface potential under the applied gate voltage, Poisson’s equation can be employed:

(14) $$\frac{d^{2}y\left(x\right)}{dx^{2}}=\frac{q\cdot n(y)}{k_{s}\cdot \varepsilon_{0}}$$

where k_s_ and ε_0_ are the dielectric constant of the IZO semiconductor and permittivity of vacuum, respectively. By considering the electric field at a distance x from the semiconductor gate dielectric interface and utilizing the following definition, the relationship between the electric field and the induced carrier density can be derived.

(15) $$\xi \left(x\right)=-\frac{dy\left(x\right)}{dx},\frac{d^{2}y\left(x\right)}{dx^{2}}=\frac{1}{2}\frac{d}{dy}{\left(\frac{dy\left(x\right)}{dx}\right)}^{2}$$

By integrating Poisson’s equation from x to the surface using Equation (15), the following expression is obtained:

(16) $$\frac{dy\left(x\right)}{dx}=-{\left(\frac{2\cdot e}{k_{s}\cdot \varepsilon_{0}}\int_{0}^{y\left(x\right)}n\left(y\right)dy\right)}^{1/2}$$

The boundary conditions applied were y(d_s_) = 0 and dy(d_s_)/dx = 0 at the top of the semiconductor surface d_s_, and y(0) = V_F_ and dy(x)/dx = −ξ_s_(x). Substituting x with y as a function of Equation (13), under the dy_s_/dx condition in Equation (16), and rearranging according to I_D_(V_GS_)/I_FB_ − 1, we can express it as:

(17) $$\frac{I_{D}\left(V_{G}\right)-I_{FB}}{I_{FB}}=\frac{1}{d_{s}}\cdot \int_{0}^{y_{s}}\frac{\exp\left[\left(\beta -A\right)\cdot y\left(x\right)\right]-1}{{\left(\frac{2\cdot e}{k_{s}\cdot \varepsilon_{0}}\int_{0}^{y\left(x\right)}n\left(y\right)dy\right)}^{1/2}}dy$$

The induced carriers are electrons; therefore, a negative sign is applied to the relationship between the electric field and the carriers. The relationship between the electric field and the gate bias is expressed as follows:

(18) $$k_{s}\cdot E\left(0\right)=-k_{s}\cdot \frac{dy_{s}}{dx}=k_{ins}\cdot \frac{V_{GS}-V_{Th}-y_{s}}{d_{ins}}$$

where k_ins_ is the dielectric constant of the gate dielectric, and d_ins_ is its thickness. To simplify the calculation, we assume that y_s_ is much smaller than V_GS_ − V_Th_. The applied voltage V_F_ follows the previously mentioned V_F_(V_GS_) = V_GS_ − V_Th_(V_GS_) condition from a simple charge approximation. From Equation (18), V_F_ can be expressed as follows:

(19) $$V_{G}-V_{Th}(V_{G})\equiv V_{F}=-\frac{k_{s}\cdot d_{ins}}{k_{ins}}\cdot \frac{dy_{s}}{dx}=\frac{k_{s}\cdot d_{ins}}{k_{ins}}{\left(\frac{2\cdot q}{k_{s}\cdot \varepsilon_{0}}\int_{0}^{y_{s}}n\left(y\right)dy\right)}^{1/2}.$$

Differentiating the above equation with respect to y_s_, we obtain:

(20) $$\frac{{dV}_{F}}{{dy}_{s}}=\frac{k_{s}\cdot d_{ins}}{k_{ins}}\cdot {\left(\frac{q}{{2\cdot k}_{s}\cdot \varepsilon_{0}}\right)}^{\frac{1}{2}}\cdot {\left(\int_{0}^{y_{s}}n\left(y\right)dy\right)}^{-1/2}\cdot n\left(y_{s}\right).$$

By examining the relationship between y_s_ and V_F_, substituting Equation (18) into Equation (17), and differentiating with respect to V_F_, we obtain the equation for transconductance:

(21) $$\frac{1}{I_{FB}}\cdot \frac{{dI}_{D}}{{dV}_{F}}=\frac{1}{d_{s}}\cdot \frac{\exp\left[\left(\beta -A\right)\cdot y_{s}\right]-1}{{\left(\frac{2\cdot e}{k_{s}\cdot \varepsilon_{0}}\int_{0}^{y_{s}}n\left(y\right)dy\right)}^{1/2}}\cdot \frac{dy_{s}}{dV_{F}}=\frac{\exp\left[\left(\beta -A\right)\cdot y_{s}\right]-1}{\left(\frac{k_{s}\cdot d_{ins}}{k_{ins}}\right)\cdot d_{s}\cdot \left(\frac{e}{k_{s}\cdot \varepsilon_{0}}\right)\cdot n\left(y_{s}\right)}.$$

By rearranging the above equation for the carrier density n(y_s_), we can obtain the relationship between transconductance dI_D_/dV_F_ and n(y_s_).

(22) $$n\left(y_{s}\right)=\frac{k_{ins}\cdot \varepsilon_{0}}{q\cdot d_{ins}\cdot d_{s}}\cdot \frac{I_{FB}\cdot \left\{\exp\left[\left(\beta -A\right)\cdot y_{s}\right]-1\right\}}{dI_{D}/dV_{F}}$$

To extract the surface energy band bending y_s_ from the relationship between V_F_ and y_s_, we rearrange Equation (21) for dV_s_/dV_F_ and substitute Equation (18), which results in the following expression:

(23) $$\frac{{dy}_{s}}{{dV}_{F}}=\frac{k_{ins}}{k_{s}\cdot d_{ins}}\cdot \frac{V_{F}}{I_{FB}}\cdot \frac{{dI}_{D}}{{dV}_{F}}\cdot \frac{1}{\exp\left[\left(\beta -A\right)\cdot y_{s}\right]-1}$$

Integrating and rearranging the above equation yields:

(24) $$\exp\left[\left(\beta -A\right)\cdot y_{s}\left(V_{F}\right)\right]-\left(\beta -A\right)\cdot y_{s}\left(V_{F}\right)-1=\frac{\beta -A}{I_{FB}}\cdot \frac{d_{s}}{d_{ins}}\cdot \frac{k_{ins}}{k_{s}}\cdot \left[V_{F}\cdot I_{D}\left(V_{F}\right)-\int_{0}^{V_{F}}I_{D}\left(V_{F}^{\prime }\right)dV_{F}^{\prime }\right]$$

Equation (24) is a nonlinear equation without a solution. The solution for y_s_ on the left side can be approximated using an iteration based on the results obtained by substituting V_F_ and I_D_(V_F_) on the right side. Using the calculations performed thus far, the final distribution of the acceptor-like state density N(E) can be obtained as follows:

(25) $$N\left(E\right)={\left|\frac{dn\left(y_{s}\right)}{dy_{S}}\right|}_{y_{s}=E},N\left(E\right)=N_{C}\exp\left(-\frac{E_{C}-E}{kT_{c}}\right).$$

**Figure 8:**
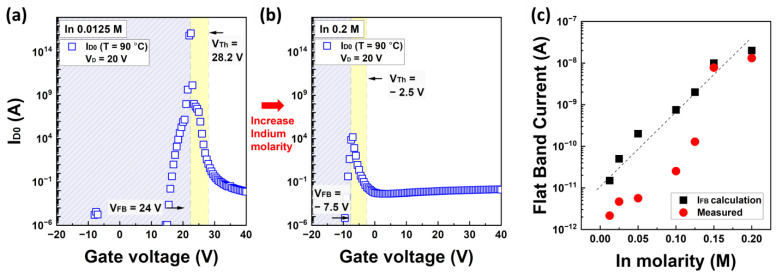
The MN prefactor I_D0_ graph as a function of the gate voltage (**a**) at 0.0125 M of In molarity and (**b**) at 0.2 M of In molarity. The yellow region in graphs (**a**,**b**) corresponds to the subthreshold voltage region. (**c**) The estimated and measured flat band current characteristics in accordance with the In molarity ratio.

Figure 8 shows the MN prefactor I_D0_ and flat-band current values as functions of the applied gate voltage in the IZO TFTs. More detailed results for I_D0_ as a function of the In concentration are provided in the Supplementary Materials (Figure S7). In Figure 8a,b, V_FB_ and V_Th_ are determined using a simple charge approximation. The hatched region represents the depletion region and the yellow box indicates the subthreshold region. The speculated value of I_FB_ in Figure 8c is inferred from the off-state current in the transfer characteristics. While an approximate value of I_FB_ was estimated from the transfer characteristics, the I_FB_ values shown in Figure 8c are approximations obtained through the calculations in Equations (22) and (24). As shown in Figure 8a,b, the approximate MN prefactor I_D0_ exhibits an inverse relationship with the gate voltage and decreases significantly with increasing In molarity ratio.

**Figure 9:**
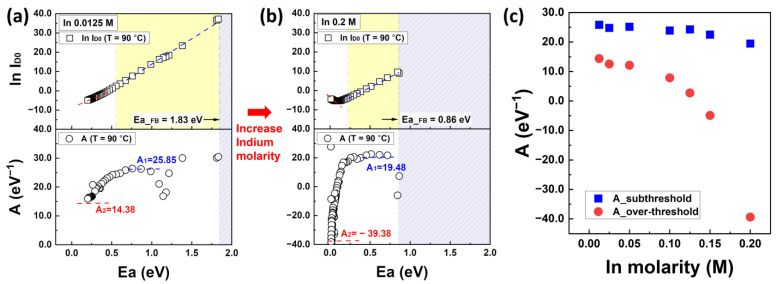
The characteristics of the MN prefactor and MN parameter A graph depending on the activation energy at (**a**) 0.0125 M of In molarity ratio and (**b**) 0.2 M of In molarity ratio. The yellow region represents the E_a_ in the subthreshold voltage region. (**c**) The MN parameter A in the subthreshold voltage region and overthreshold voltage region with respect to the In molarity ratio.

Figure 9 shows the ln I_D0_ graph as a function of the activation energy and MN parameter A with respect to the In molarity ratio. The complete extraction results of MN parameter A for the entire In concentration range are summarized in Supplementary Material Figure S8. The MN parameter A in Figure 9a,b indicates the slopes obtained by differentiating ln I_D0_ in terms of E_a_ and can be defined in two regions. The region where I_D0_ corresponds to the subthreshold region is depicted within the yellow box, whereas the region from the edge of the yellow box to 0 eV represents the overthreshold voltage region. Results: A in the two regions was defined based on the average slopes in each region as A__subthreshold_ and A__overthreshold_. A1 and A2 in Figure 9a,b refer to A__subthreshold_ and A__overthreshold_, respectively. As mentioned earlier, the value of E_a_ in Equation (11) is a function of bias and temperature. Since we cannot simultaneously use two variables in a single equation like Equation (12), we will specify the value of A to incorporate the temperature factor. In this case, the A__subthreshold_ value represents the influence at low temperatures, while the A__overthreshold_ value represents the influence at high temperatures. By doing so, we can specify the thermal energy at low and high temperatures and ultimately calculate the shallow/deep state distribution based on the bias. The atypical magnitude of negative A, especially at high temperatures and high In concentrations in Figure 9b, can be attributed to the electrical behavior of a slight drain current decrease in the saturation region, which is associated with the percolation theory. As shown in Figure 9c, depending on the In molarity ratio, A__subthreshold_ in the subthreshold region shows relatively little variation, ranging from 25.85 to 19.48 eV^−1^. However, A__overthreshold_ exhibits significant variation, ranging from 14.38 to −39.38 eV^−1^ in the overthreshold voltage region. It is important to note that, while the MN parameter A was estimated from Figure 9a,b, the exact values of A were subsequently obtained based on the calculations.

**Figure 10:**
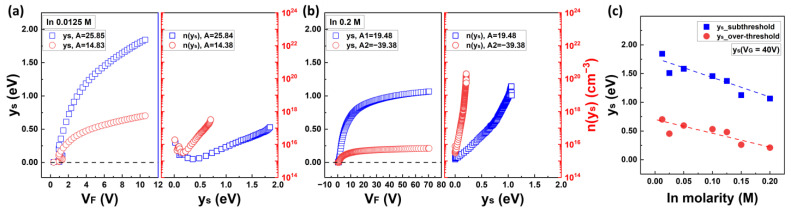
Characteristics of surface band bending and free carrier density depending on the applied voltage and surface bending, respectively. (**a**) The characteristics of solution-processed IZO TFT with 0.0125 M of In molarity and (**b**) with 0.2 M. (**c**) The maximum surface band bending at the subthreshold voltage region and overthreshold voltage region with respect to the In molarity ratio.

Figure 10 shows the surface band bending energy y_s_ as a function of the applied gate voltage V_F_, the surface free carrier density n(y_s_) in terms of y_s_, and the maximum surface band bending of y_s_ with respect to the In molarity ratio. Moreover, detailed analysis results regarding the In molarity ratio can be found in Figure S9 of the Supplementary Materials. The blue squares in Figure 10a,b represent the characteristics in the subthreshold voltage region, whereas the red circles represent the characteristics in the overthreshold voltage region. The y_s_–V_F_ graph was derived using Equation (24), and the n(y_s_)–y_s_ graph was extracted using Equation (22). The values of y_s_ obtained from Equation (24) were iteratively derived until an error of 0.1% was achieved. Furthermore, the modified I_FB_ value, I_FB_’ = 100 × I_FB_, was used in Equation (23). The interpretation of the corrected results was based on the analysis of E_a_FB_ using a simple charge approximation. Without using a correction factor of 100, y_s_ for low In, 0.0125 M, changed from 1.845 to 2.597 eV, and y_s_ for high In, 0.2 M, changed from 1.066 eV to 1.435 eV. Furthermore, if the uncorrected I_FB_ were applied, the carrier density n(y_s_) at the degenerated states would increase by a factor of 10^2^, resulting in the DOS at the conductor band edge N_C_ reaching levels as high as 10^25^ cm^−3^·eV^−1^. Using the correction factor, the interpretation of the IZO semiconductor characteristics can be considered theoretically reasonable. As shown in Figure 10c, for an applied gate voltage of V_G_ = 40 V, the maximum surface band bending y_s_ decreased with increasing In molarity ratio and closely resembled the result of E_a_FB_ in the subthreshold region as depicted in Figure 3c. The characteristics of y_s_ in the region above the threshold voltage can be understood as a decrease in activation energy with a significant amount of thermal energy. This thermal energy, which is represented by the MN parameter A in the equation, plays a role in reducing the activation energy during the TFT operation.

**Figure 11:**
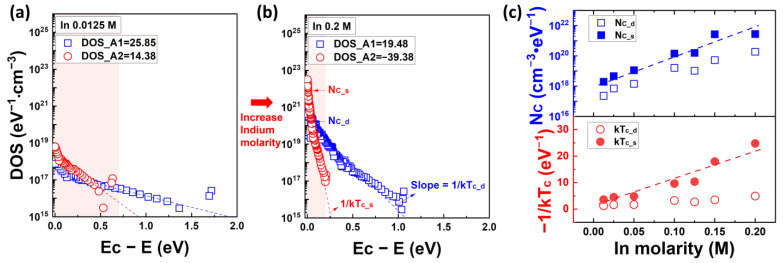
Extracted acceptor-like state distribution using MN Rule-based field-effect analysis. (**a**) The DOS profile and fitting model of shallow/deep states at In 0.0125 M and (**b**) at In 0.2 M. The red area in graphs (**a**,**b**) represents the DOS in the overthreshold voltage region. (**c**) Density of shallow/deep states at the edge of the conduction band and their characteristic temperature results.

The calculated distribution of acceptor-like states with respect to the In molarity ratio is shown in Figure 11. More detailed results on the acceptor-like state density as a function of the In molarity ratio can be found in Supplementary Figure S10. In Figure 11a,b, the blue rectangles and red circles represent the extracted DOS distributions in the subthreshold and overthreshold regions, respectively. The red box indicates the DOS characteristics in the overthreshold-voltage region. The blue and red dashed lines in the graph represent the exponential tangent lines of the shallow and deep states, respectively. The DOS concentrations at the conduction band edges, N_C_s_ and N_C_d_, correspond to the characteristics of the shallow and deep states, respectively, whereas the characteristic energies kT_c_s_ and kT_c_d_ represent the slopes of the shallow and deep state characteristics. As shown in Figure 11c, the N_C_s_ value increases from 1.93 × 10^18^ eV^−3^cm^−3^ to 2.77 × 10^21^ eV^−3^cm^−3^ with respect to the In molarity ratio, while the kT_c_s_ value decreases from approximately 280 meV to 40 meV.

In addition, the extracted N_C_ depending on the calculation methods are summarized in Table 2. As shown in Table 2, the calculated results demonstrate a similar magnitude for both approaches. However, it’s important to note that the simple charge approximation method may lead to inaccuracies and fluctuation, especially in the deep state region. This is attributed to drawing two tangents from the one distribution in the calculation. On the other hand, the advantage of the MN Rule-based field-effect analysis over the charge sheet approximation method is its theoretical foundation and accuracy based on parameters such as the conductivity dI_D_/dV_F_ with respect to the gate voltage. It provides a more detailed DOS distribution from the deep to the shallow states. This is because MN constant, A, has been appropriately characterized into subthreshold and overthreshold regions. As a result, the extracted DOS distribution characteristics obtained from the two methods were similar, with an exponential increase in N_C_ and a linear decrease in E_a_FB_ with respect to the In molarity ratio. As mentioned above in the atomic bonding structure model, the atomic bonding structure of Zn–O or In–O is determined by factors such as the charge density of metal cations and the atomic sizes. In case of solution-processed IZO semiconductors, the amorphous random network structure is determined by Zn–O bonding, and depending on the In concentration In atoms replace Zn atoms. The enhanced electrical conductivity of IZO semiconductors has been empirically confirmed [26,27]. By replacing the ionic bonding of Zn^2+^ with the ionic bonding of In^3+^, the In–O bonding structure can act as donor, and free electrons are generated through the reaction of the dangling bond D_InO_^−^ → D_InO_^0^ + e^−^. These free electrons can improve the conductivity of the IZO semiconductor. Based on the fundamentals of the solution-processed IZO semiconductor, the DOS distributions were calculated quantitatively, revealing an increase in weak, dangling bonds and oxygen vacancies within the In–O atomic bonding structure. This led to a significant increase in the number of donors near the conduction band edge. The DOS extraction method presented in this study is applicable to a wide range of amorphous semiconductor materials and is effective in predicting the precise position of the Fermi energy. This provides a versatile approach that can be employed to understand the electronic properties and device performances of various material systems.

## 5. Conclusions

In conclusion, this study focused on the acceptor-like state profile of solution-processed IZO semiconductors using simple charge approximation and MN rule-based field-effect analysis. The quantitative estimation of the DOS profiles further reveals an increase in weak bonds, dangling bonds and oxygen vacancies in the InO atomic bonding structures with higher In molarity ratios. This led to a significant increase in the donor-state concentration near the conduction band edge. The increase in donor with respect to the In molarity is interpreted as the result of In atoms replacing Zn atoms in the base of the Zn–O amorphous random network. This change in the bonding structure is attributed to the differences in charge density and atomic size between In and Zn atoms. The reliability and accuracy of the proposed approach were validated by comparing the DOS profiles extracted using this approach with those obtained using a simple charge approximation method. These results highlight the importance of understanding the effect of the In molarity ratio on the acceptor-like state distribution. The results demonstrated that the acceptor-like state distribution varied significantly with the In molarity ratio, indicating material property dependence on the composition. Moreover, calculation of the quantitative profile enables precise prediction of the Fermi energy position, facilitating the design and optimization of solution-processed IZO TFTs. The obtained results not only contribute to the understanding of material properties but also have practical implications. In particular, the temperature-dependent characteristics of solution-processed IZO TFT will be utilized in future research on topics such as field-effect mobility modeling, and can also be compared with TCAD simulation results. The practical approach and detailed results obtained in this study provide valuable insights for the development of reliable and efficient electronic devices using amorphous semiconductors. 

## Figures and Tables

**Table 1:** The molarity ratio of the IZO solution with respect to the In concentration.

No.	In01	In02	In03	In04	In05	In06	In07	In08
Zn molarity (M)	0.25 (fixed)							
In molarity (M)	0 (non-operational)	0.0125	0.025	0.05	0.1	0.125	0.15	0.2
In, Zn atomic weight ratio, In/Zn	0	0.086	0.178	0.350	0.706	0.883	1.055	1.368

**Table 2:** DOS at the conduction band edge as a function of In molarity ratio.

No.	N_C_ (cm^−3^·eV^−1^)	In 0.0125 M	In 0.025 M	In 0.05 M	In 0.1 M	In 0.125 M	In 0.15 M	In 0.2 M
Simple charge approximation	N_C_tail_	9.59 × 10^18^	1.02 × 10^20^	8.01 × 10^19^	1.51 × 10^20^	1.53 × 10^21^	2.00 × 10^22^	7.63 × 10^21^
	N_C_deep_	3.48 × 10^18^	4.19 × 10^18^	8.37 × 10^18^	3.42 × 10^18^	1.30 × 10^18^	2.60 × 10^19^	1.10 × 10^19^
MN Rule field- effect analysis	N_C_s_	1.93 × 10^18^	4.57 × 10^18^	1.12 × 10^19^	1.41 × 10^20^	1.57 × 10^20^	2.68 × 10^21^	2.76 × 10^21^
	N_C_d_	2.28 × 10^17^	7.03 × 10^17^	1.44 × 10^18^	1.61 × 10^19^	1.05 × 10^19^	5.25 × 10^19^	1.87 × 10^20^

## Data Availability

The research data presented in this study are available on request from the corresponding author.

## Supplementary Materials

The following supporting information can be downloaded at: https://www.mdpi.com/article/10.3390/nano13152165/s1, Figure S1. (a–g) Transfer characteristics of solution-processed IZO TFTs at various temperatures. All IZO TFTs in the graphs were fabricated using a ZnO solution with a molarity of 0.25 M, while the In molarities in (a–g) correspond to 0.0125, 0.025, 0.05, 0.1, 0.125, 0.15, and 0.2 M, respectively. (h) On-state current of IZO TFTs as a function of the In molarity ratio. Figure S2. (a–g) Arrhenius plots of IZO TFTs depending on the In molarity ratio. (h) Activation energy as a function of gate voltage at T = 240 °C, shown for different In molarity ratios. Figure S3. (a–g) Activation energy versus gate voltage graph for different In molarity ratios. (h) illustrates the variation in activation energy and flat band voltage depending on the In molarity ratio. Figure S4. (a–g) V_G_-sqrt(I_D_) graphs for extracting the threshold voltage of solution-processed IZO TFTs. (a–g) Graphs represent the results for different In molarity ratios, with the yellow region indicating the subthreshold voltage region. (h) V_Th_ results for various In molarity ratios are shown at RT and T = 90 °C. Figure S5. Field-effect mobility, µ_FE_, characteristics as a function of gate voltage with respect to the In molarity ratio. Figure S6. (a–g) The DOS distribution calculated using the simple charge approximation method. Each (a–g) graph corresponds to a different In molarity ratio, and the tangent lines represent the exponential distribution models of the shallow (band tail) states and deep states, respectively. (h) Graph showing the variation in N_C_ and −1/kT_c_ with respect to the In molarity ratio. N_C_ represents the DOS value at E_C_, and T_C_ represents the characteristic temperature. Figure S7. (a–g) Graphs of the MN prefactor (I_D0_) as a function of gate voltage. Each graph represents the results for different In molarity ratios, and the hatched area and yellow area correspond to the region below the flat band voltage and the subthreshold voltage region, respectively. (h) Flat band current values according to the In molarity ratio. The flat band current values were extracted from the off-state current of the transfer curves. Figure S8. (a–g) The In(I_D0_) and MN constant, A, as a function of activation energy. Each graph represents the characteristics for different In molarity ratios. (h) The MN constant, A, in the subthreshold voltage region and overthreshold voltage region with respect to the In molarity ratio. Figure S9. (a–g) Graphs of V_F_–y_s_ and y_s_–n(y_s_) as a function of In molarity ratio. The red y-axis represents the values of n(y_s_). (h) The maximum value of y_s_ at V_G_ = 40 V as a function of In molarity ratios. Figure S10. (a–g) The DOS distribution calculated using the MN rule-based field-effect analysis method. (a–e) represent the graphs for different In molarity ratios. The tangent lines in the graphs depict the exponential distribution models for shallow states and deep states. (h) Graphs showing the characteristics of shallow states and deep states, represented by N_C_ and kT_c_, respectively, as a function of In molarity ratio.
