# Supplementary material for: Analyzing Acceptor-like State Distribution of Solution-Processed Indium-Zinc-Oxide Semiconductor Depending on the In Concentration
Source: Nanomaterials (Basel). 2023 Jul 26;13(15):2165. doi: 10.3390/nano13152165 (PMC10421299; doi:10.3390/nano13152165)
Supplement: Supplementary file 1 [file nanomaterials-13-02165-s001.zip › nanomaterials-2499502-supplementary.pdf]

Figure S1. (a-g) Transfer characteristics of solution-processed IZO TFT at various temperatures. All IZO TFTs in the graphs were fabricated using a ZnO solution with a molarity of 0.25 M, while the In molarities in (a-g) correspond to 0.0125, 0.025, 0.05, 0.1, 0.125, 0.15, and 0.2 M, respectively. (h) On-state current of IZO TFTs as a function of the In molarity ratio.

Figure S2. (a-g) Arrhenius plots of IZO TFTs depending on the In molarity ratio. (h) Activation energy as a function of gate voltage at T = 240 °C, shown for different In molarity ratios.

Figure S3. (a-g) Activation energy versus gate voltage graph for different In molarity ratios. (h) illustrates the variation of activation energy and flat band voltage depending on the In molarity ratio.

Figure S4. (a-g) V_G-sqrt(I_D) graphs for extracting the threshold voltage of solution-processed IZO TFTs. (a-g) graph represents the results for different In molarity ratios, with the yellow region indicating the subthreshold voltage region. The black dashed line represents the tangent at $V_{G.max}$, whereas the red dashed line represents the tangent at the gate voltage near V_{FB}. (h) V_{Th} results for various In molarity ratios are shown at RT and T = 90 °C.

Figure S5. Field-effect mobility, μ_{FE}, characteristics as a function of gate voltage with respect to the In molarity ratio.

Figure S6. (a-g) The DOS distribution calculated using the simple charge approximation method. Each (a-g) graph corresponds to a different In molarity ratio, and the tangent lines represent the exponential distribution models of the shallow (band tail) states and deep states, respectively. (h) Graph showing the variation of N_C and $-1/kT_c$ with respect to the In molarity ratio. N_C represents the DOS value at E_C, and T_C represents the characteristic temperature.

Figure S7. (a-g) Graphs of the MN prefactor (I_{D0}) as a function of gate voltage. Each graph represents the results for different In molarity ratios, and the hatched area and yellow area correspond to the region below the flat band voltage and the subthreshold voltage region, respectively. (h) Flat band current values according to the In molarity ratio. The flat band current values were extracted from the off-state current of the transfer curves.

Figure S8. (a-g) The In(I_{D0}) and MN constant, A as a function of activation energy. Each graph represents the characteristics for different In molarity ratios. (h) The MN constant, A in the subthreshold voltage region and over-threshold voltage region with respect to the In molarity ratio.

Figure S9. (a-g) Graphs of V_F–y_s and y_s–n(y_s) as a function of In molarity ratio. The red y-axis

represents the values of $n(y_s)$. (h) The maximum value of y_s at V_G = 40 V as a function of In molarity ratios.

Figure S10. (a-g) The DOS distribution calculated using the MN-rule-based field-effect analysis method. (a-e) represent the graphs for different In molarity ratios. The tangent lines in the graphs depicted the exponential distribution models for shallow states and deep states. (h) Graphs showing the characteristics of shallow states and deep states, represented by N_C and kT_c, respectively, as a function of In molarity ratio.

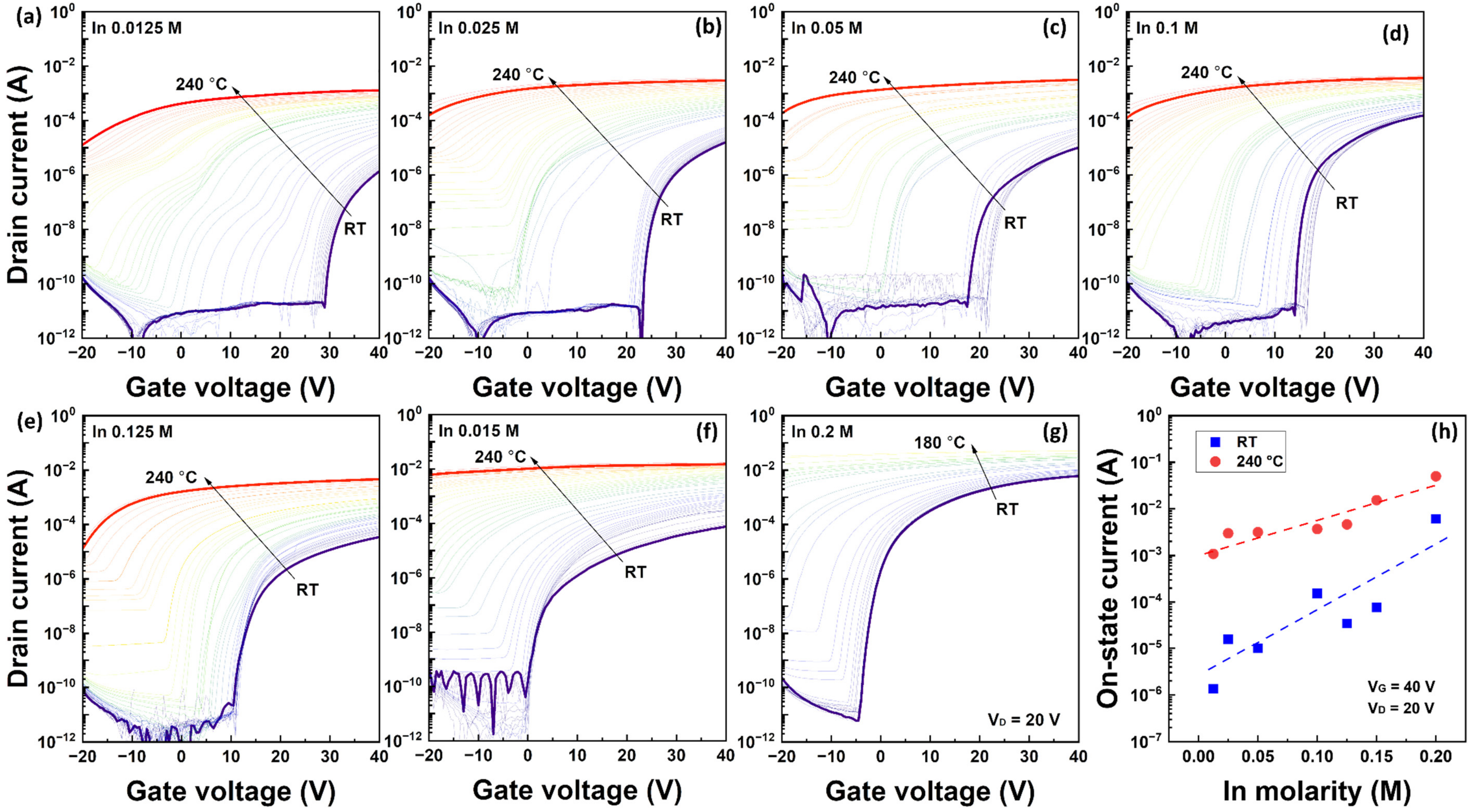

(a)
In 0.0125 M
240 °C
RT
Drain current (A)
Gate voltage (V)
(b)
In 0.025 M
240 °C
RT
Gate voltage (V)
(c)
In 0.05 M
240 °C
RT
Gate voltage (V)
(d)
In 0.1 M
240 °C
RT
Gate voltage (V)
(e)
In 0.125 M
240 °C
RT
Drain current (A)
Gate voltage (V)
(f)
In 0.015 M
240 °C
RT
Gate voltage (V)
(g)
In 0.2 M
180 °C
RT
VD = 20 V
Gate voltage (V)
(h)
RT
240 °C
VG = 40 V
VD = 20 V
On-state current (A)
In molarity (M)

(Figure S1)

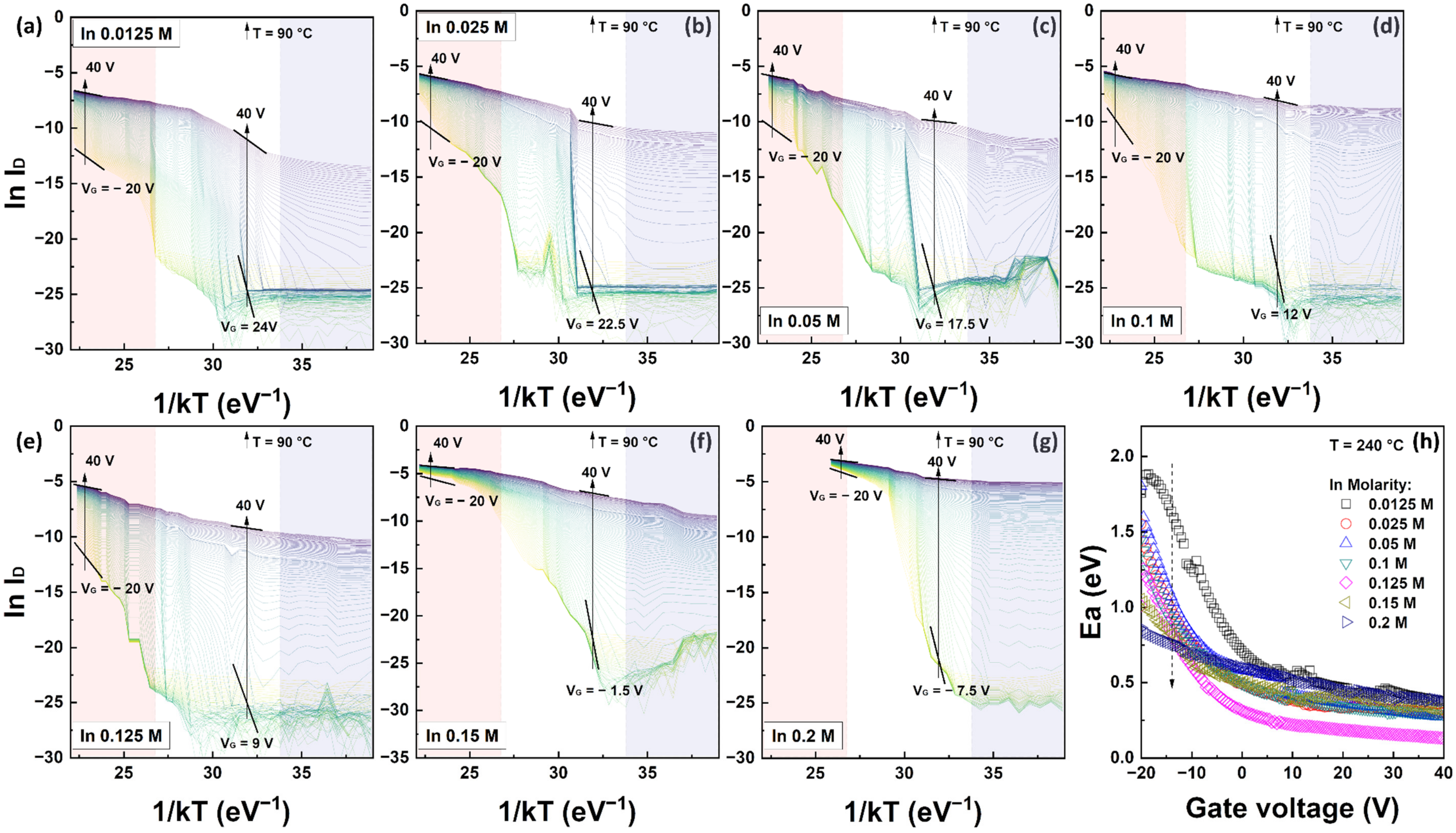
(a)
In 0.0125 M
T = 90 °C
40 V
V_G = − 20 V
40 V
V_G = 24V
ln I_D
1/kT (eV^{-1})
(b)
In 0.025 M
T = 90 °C
40 V
V_G = − 20 V
40 V
V_G = 22.5 V
1/kT (eV^{-1})
(c)
In 0.05 M
T = 90 °C
40 V
V_G = − 20 V
40 V
V_G = 17.5 V
1/kT (eV^{-1})
(d)
In 0.1 M
T = 90 °C
40 V
V_G = − 20 V
40 V
V_G = 12 V
1/kT (eV^{-1})
(e)
In 0.125 M
T = 90 °C
40 V
V_G = − 20 V
40 V
V_G = 9 V
ln I_D
1/kT (eV^{-1})
(f)
In 0.15 M
T = 90 °C
40 V
V_G = − 20 V
40 V
V_G = − 1.5 V
1/kT (eV^{-1})
(g)
In 0.2 M
T = 90 °C
40 V
V_G = − 20 V
40 V
V_G = − 7.5 V
1/kT (eV^{-1})
(h)
T = 240 °C
In Molarity:
0.0125 M
0.025 M
0.05 M
0.1 M
0.125 M
0.15 M
0.2 M
Ea (eV)
Gate voltage (V)

(Figure S2)

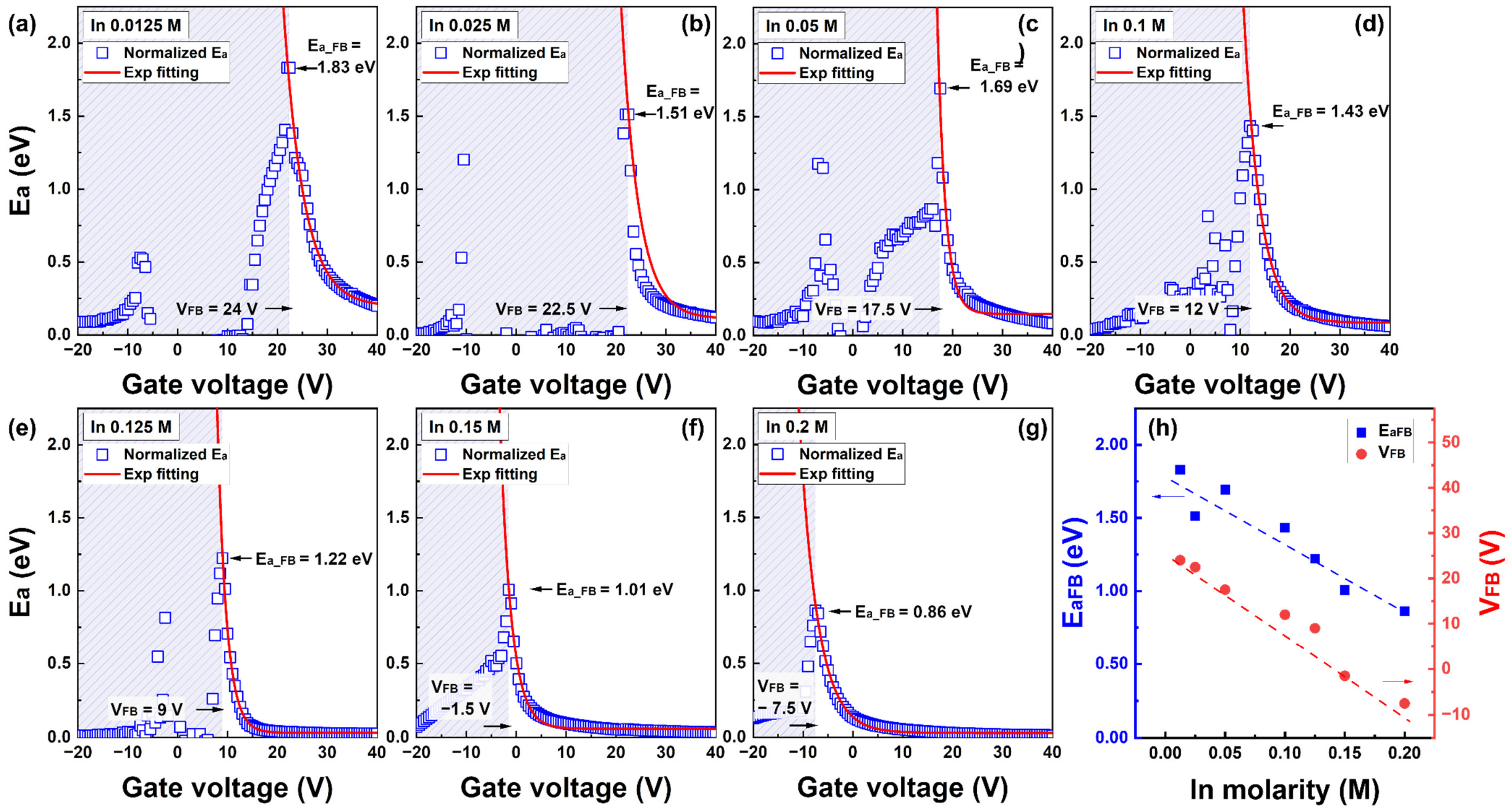

(a)
In 0.0125 M
Normalized Ea
Exp fitting
Ea_FB = 1.83 eV
VFB = 24 V
(b)
In 0.025 M
Ea_FB = 1.51 eV
VFB = 22.5 V
(c)
In 0.05 M
Ea_FB = 1.69 eV
VFB = 17.5 V
(d)
In 0.1 M
Ea_FB = 1.43 eV
VFB = 12 V
(e)
In 0.125 M
Ea_FB = 1.22 eV
VFB = 9 V
(f)
In 0.15 M
Ea_FB = 1.01 eV
VFB = −1.5 V
(g)
In 0.2 M
Ea_FB = 0.86 eV
VFB = − 7.5 V
Ea (eV)
Gate voltage (V)
(h)
EaFB
VFB
EaFB (eV)
VFB (V)
In molarity (M)

(Figure S3)

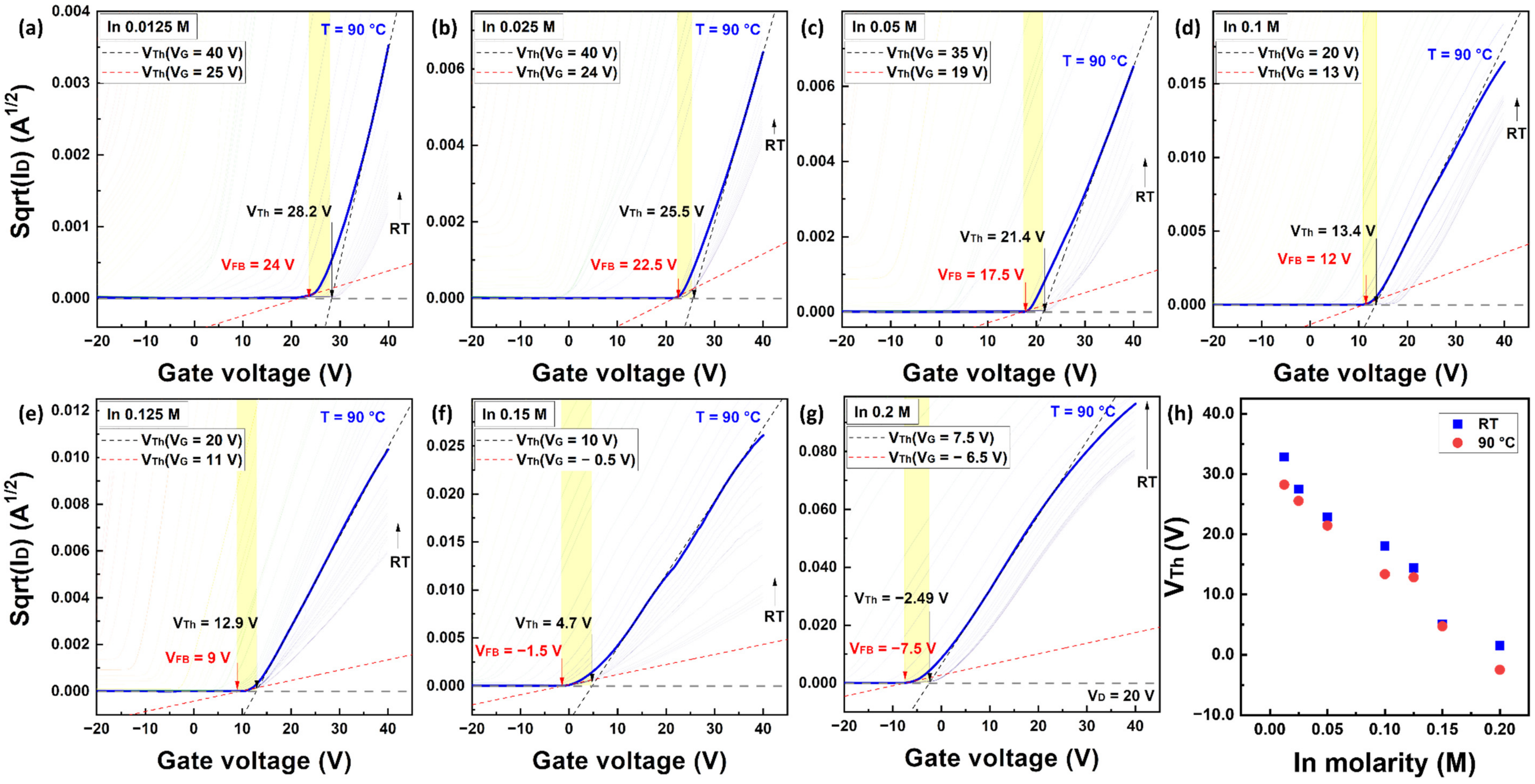

(a)
In 0.0125 M
VTh(VG = 40 V)
VTh(VG = 25 V)
T = 90 °C
VTh = 28.2 V
VFB = 24 V
RT
(b)
In 0.025 M
VTh(VG = 40 V)
VTh(VG = 24 V)
T = 90 °C
VTh = 25.5 V
VFB = 22.5 V
RT
(c)
In 0.05 M
VTh(VG = 35 V)
VTh(VG = 19 V)
T = 90 °C
VTh = 21.4 V
VFB = 17.5 V
RT
(d)
In 0.1 M
VTh(VG = 20 V)
VTh(VG = 13 V)
T = 90 °C
VTh = 13.4 V
VFB = 12 V
RT
(e)
In 0.125 M
VTh(VG = 20 V)
VTh(VG = 11 V)
T = 90 °C
VTh = 12.9 V
VFB = 9 V
RT
(f)
In 0.15 M
VTh(VG = 10 V)
VTh(VG = − 0.5 V)
T = 90 °C
VTh = 4.7 V
VFB = −1.5 V
RT
(g)
In 0.2 M
VTh(VG = 7.5 V)
VTh(VG = − 6.5 V)
T = 90 °C
VTh = −2.49 V
VFB = −7.5 V
VD = 20 V
RT
(h)
RT
90 °C
Sqrt(ID) (A1/2)
Gate voltage (V)
VTh (V)
In molarity (M)

(Figure S4)

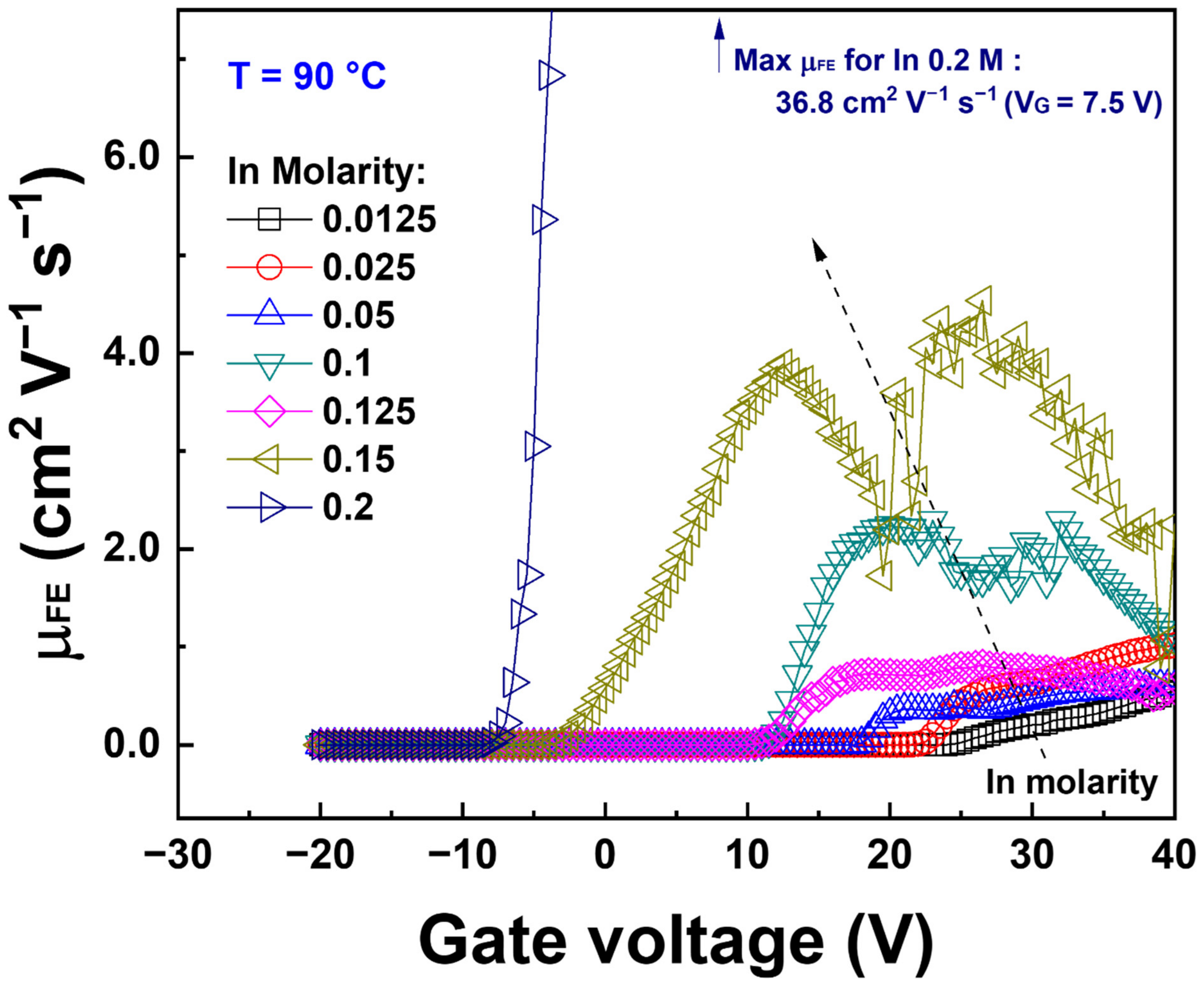

T = 90 °C
Max μFE for In 0.2 M :
36.8 cm2 V−1 s−1 (VG = 7.5 V)
In Molarity:
0.0125
0.025
0.05
0.1
0.125
0.15
0.2
In molarity
μFE (cm2 V−1 s−1)
Gate voltage (V)

(Figure S5)

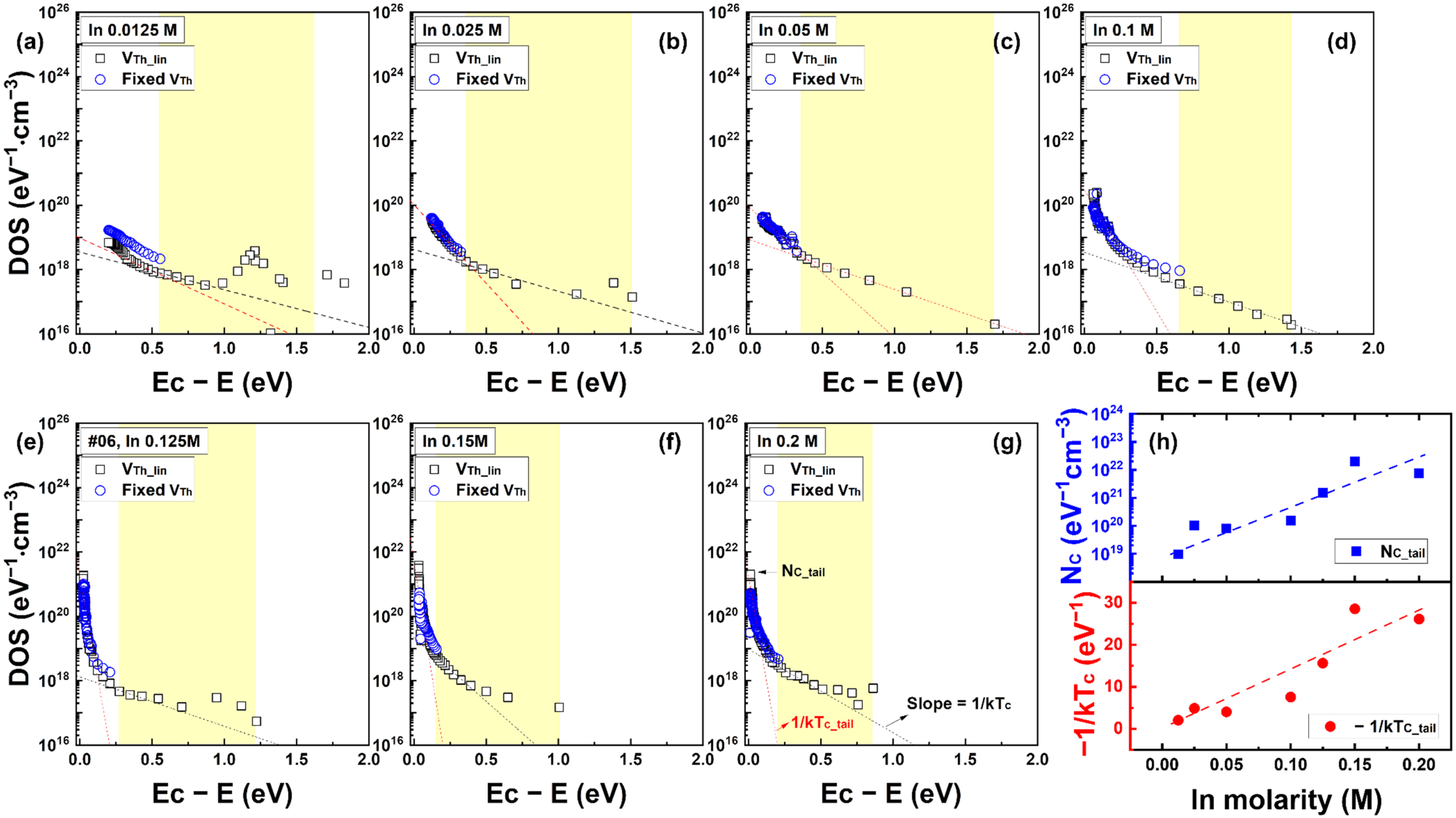

In 0.0125 M
(a)
In 0.025 M
(b)
In 0.05 M
(c)
In 0.1 M
(d)
#06, In 0.125M
(e)
In 0.15M
(f)
In 0.2 M
(g)
(h)
VTh_lin
Fixed VTh
DOS (eV−1.cm−3)
Ec − E (eV)
Nc_tail
1/kTc_tail
Slope = 1/kTc
Nc (eV−1cm−3)
−1/kTc (eV−1)
− 1/kTc_tail
In molarity (M)

(Figure S6)

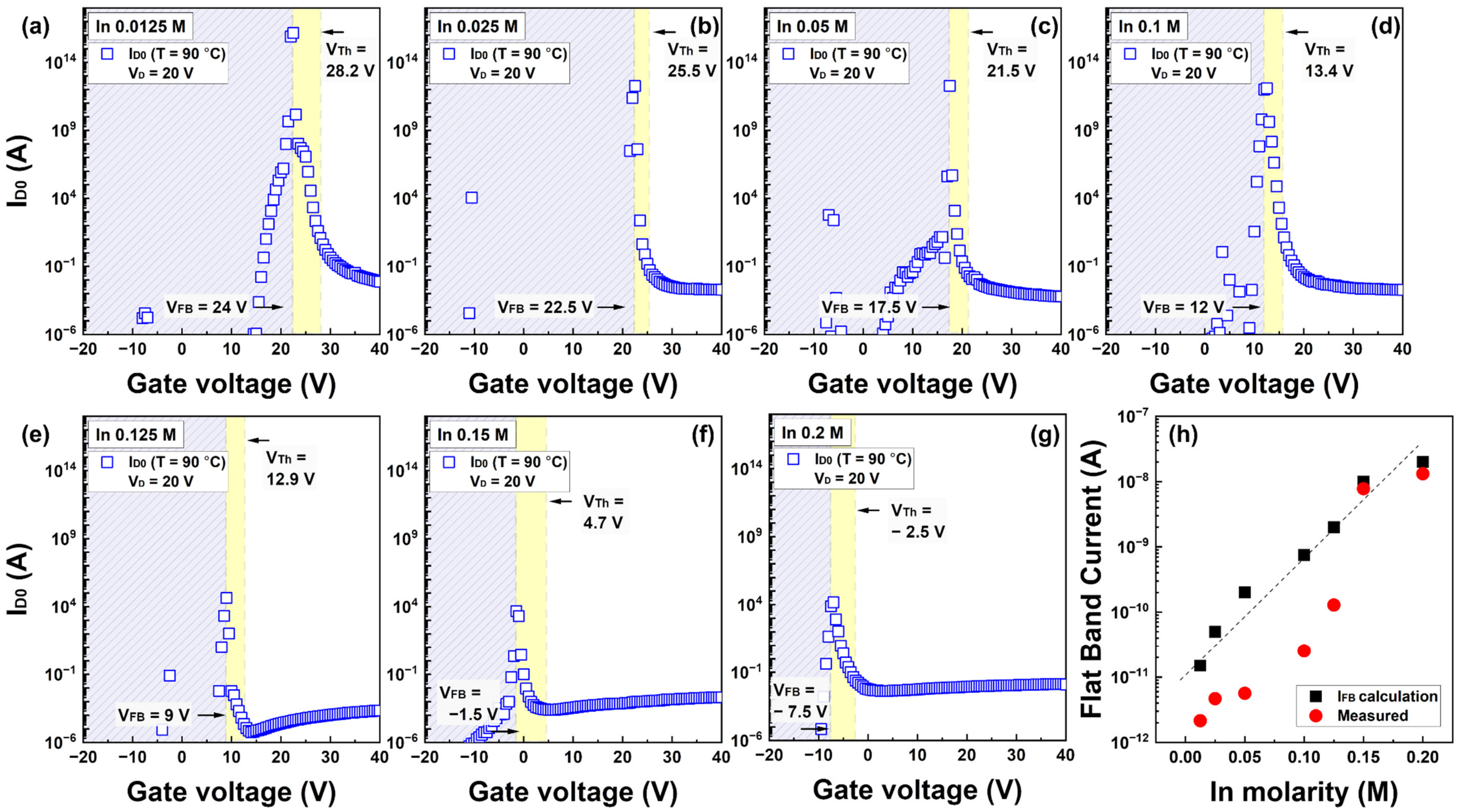

(a)
In 0.0125 M
ID0 (T = 90 °C)
VD = 20 V
VTh = 28.2 V
VFB = 24 V
(b)
In 0.025 M
VTh = 25.5 V
VFB = 22.5 V
(c)
In 0.05 M
VTh = 21.5 V
VFB = 17.5 V
(d)
In 0.1 M
VTh = 13.4 V
VFB = 12 V
(e)
In 0.125 M
VTh = 12.9 V
VFB = 9 V
(f)
In 0.15 M
VTh = 4.7 V
VFB = −1.5 V
(g)
In 0.2 M
VTh = − 2.5 V
VFB = − 7.5 V
ID0 (A)
Gate voltage (V)
(h)
Flat Band Current (A)
In molarity (M)
IFB calculation
Measured

(Figure S7)

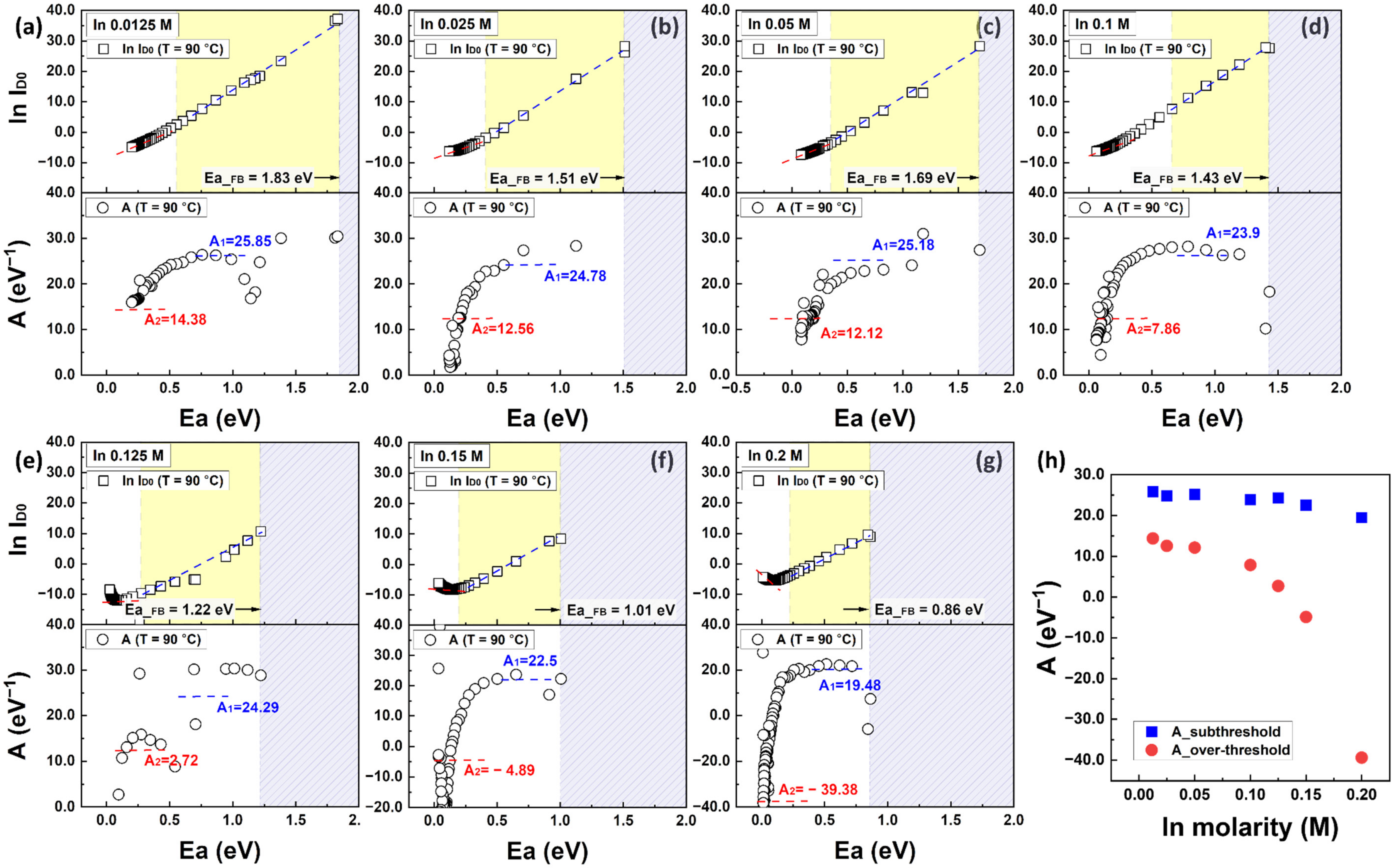

(a)
In 0.0125 M
ln ID0 (T = 90 °C)
Ea_FB = 1.83 eV
A (T = 90 °C)
A1=25.85
A2=14.38
(b)
In 0.025 M
Ea_FB = 1.51 eV
A1=24.78
A2=12.56
(c)
In 0.05 M
Ea_FB = 1.69 eV
A1=25.18
A2=12.12
(d)
In 0.1 M
Ea_FB = 1.43 eV
A1=23.9
A2=7.86
(e)
In 0.125 M
Ea_FB = 1.22 eV
A1=24.29
A2=2.72
(f)
In 0.15 M
Ea_FB = 1.01 eV
A1=22.5
A2= − 4.89
(g)
In 0.2 M
Ea_FB = 0.86 eV
A1=19.48
A2= − 39.38
(h)
A_subthreshold
A_over-threshold
ln ID0
A (eV−1)
Ea (eV)
In molarity (M)

(Figure S8)

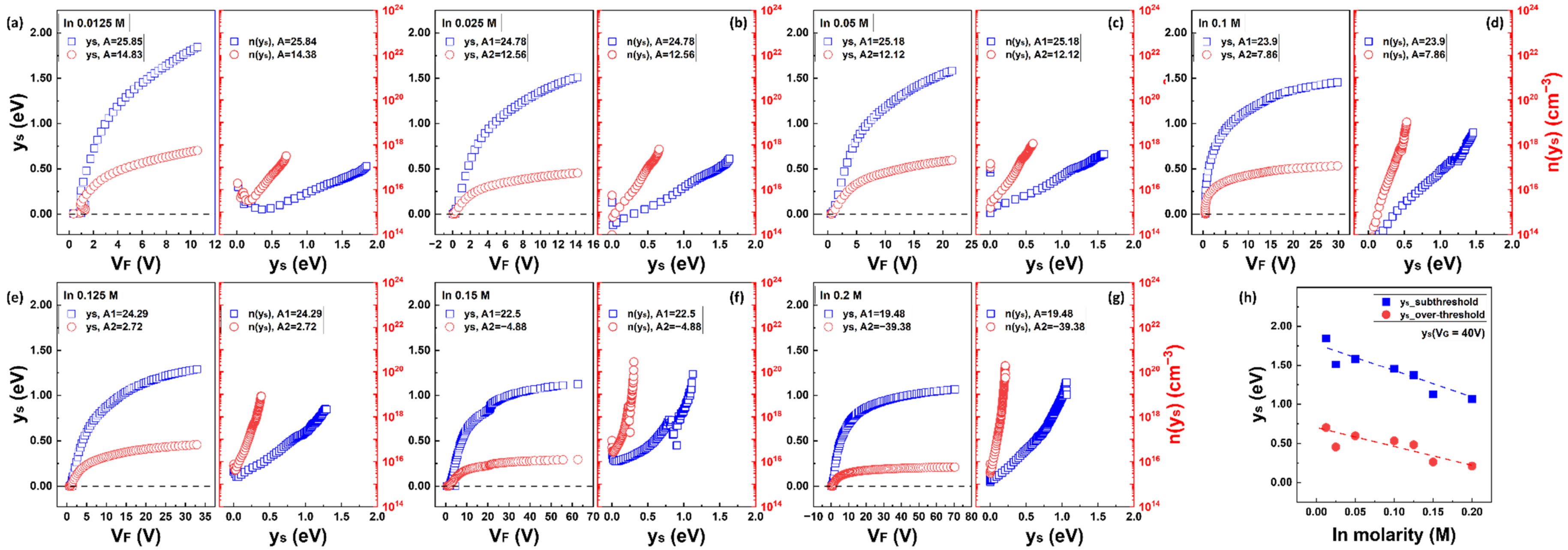
(a)
In 0.0125 M
ys, A=25.85
ys, A=14.83
n(ys), A=25.84
n(ys), A=14.38
(b)
In 0.025 M
ys, A1=24.78
ys, A2=12.56
n(ys), A=24.78
n(ys), A=12.56
(c)
In 0.05 M
ys, A1=25.18
ys, A2=12.12
n(ys), A1=25.18
n(ys), A2=12.12
(d)
In 0.1 M
ys, A1=23.9
ys, A2=7.86
n(ys), A=23.9
n(ys), A=7.86
(e)
In 0.125 M
ys, A1=24.29
ys, A2=2.72
n(ys), A1=24.29
n(ys), A2=2.72
(f)
In 0.15 M
ys, A1=22.5
ys, A2=−4.88
n(ys), A1=22.5
n(ys), A2=−4.88
(g)
In 0.2 M
ys, A1=19.48
ys, A2=−39.38
n(ys), A=19.48
n(ys), A2=−39.38
(h)
ys_subthreshold
ys_over-threshold
ys(VG = 40V)
ys (eV)
VF (V)
n(ys) (cm−3)
In molarity (M)

(Figure S9)

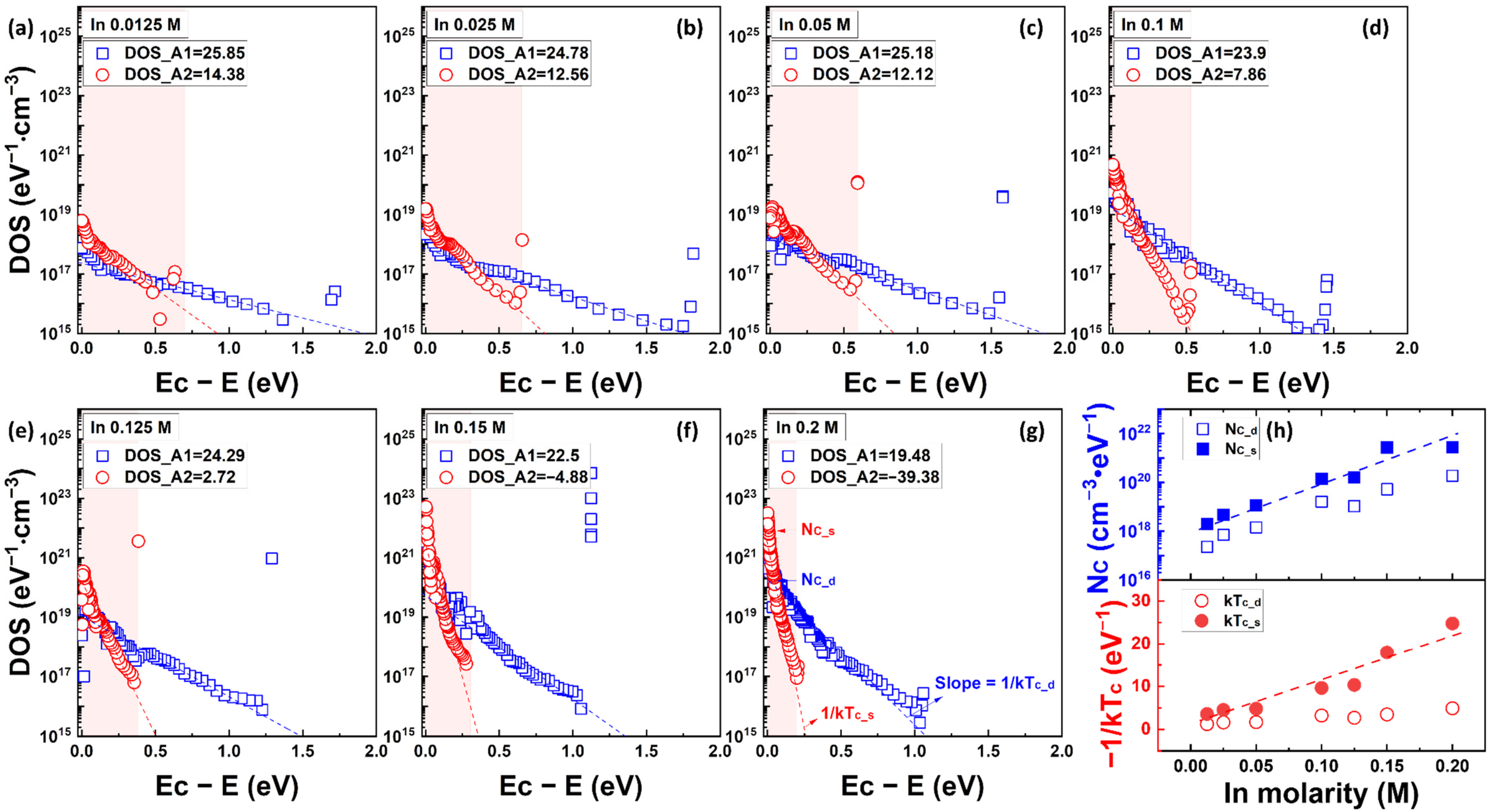

(a)
In 0.0125 M
DOS_A1=25.85
DOS_A2=14.38
(b)
In 0.025 M
DOS_A1=24.78
DOS_A2=12.56
(c)
In 0.05 M
DOS_A1=25.18
DOS_A2=12.12
(d)
In 0.1 M
DOS_A1=23.9
DOS_A2=7.86
(e)
In 0.125 M
DOS_A1=24.29
DOS_A2=2.72
(f)
In 0.15 M
DOS_A1=22.5
DOS_A2=-4.88
(g)
In 0.2 M
DOS_A1=19.48
DOS_A2=-39.38
Nc_s
Nc_d
Slope = 1/kTc_d
1/kTc_s
DOS (eV⁻¹·cm⁻³)
Ec − E (eV)
(h)
Nc_d
Nc_s
kTc_d
kTc_s
Nc (cm⁻³·eV⁻¹)
−1/kTc (eV⁻¹)
In molarity (M)

(Figure S10)
